# Potential of Stimuli-Responsive In Situ Gel System for Sustained Ocular Drug Delivery: Recent Progress and Contemporary Research

**DOI:** 10.3390/polym13081340

**Published:** 2021-04-20

**Authors:** Manisha Pandey, Hira Choudhury, Azila binti Abd Aziz, Subrat Kumar Bhattamisra, Bapi Gorain, Jocelyn Sziou Ting Su, Choo Leey Tan, Woon Yee Chin, Khar Yee Yip

**Affiliations:** 1 Department of Pharmaceutical Technology, School of Pharmacy, International Medical University, Bukit Jalil, Kuala Lumpur 57000, Malaysia; 2 Centre for Bioactive Molecules and Drug Delivery, Institute for Research, Development and Innovation, International Medical University, Kuala Lumpur 57000, Malaysia; 3 Department of Chemical and Environmental Engineering, Malaysia-Japan International Institute of Technology, Universiti Teknologi Malaysia, Jalan Sultan Yahya Petra, Kuala Lumpur 54100, Malaysia; azila@ibd.utm.my; 4 Department of Life Sciences, School of Pharmacy, International Medical University, Bukit Jalil, Kuala Lumpur 57000, Malaysia; subratkumar@imu.edu.my; 5 School of Pharmacy, Faculty of Health and Medical Sciences, Taylor’s University, Subang Jaya 47500, Selangor, Malaysia; bapi.gn@gmail.com; 6 Center for Drug Delivery and Molecular Pharmacology, Faculty of Health and Medical Sciences, Taylor’s University, Subang Jaya 47500, Selangor, Malaysia; 7 Undergraduate, School of Pharmacy, International Medical University, Bukit Jalil, Kuala Lumpur 57000, Malaysia; JOCELYN.SU@student.imu.edu.my (J.S.T.S.); TAN.CHOOLEEY@student.imu.edu.my (C.L.T.); CHIN.WOONYEE@student.imu.edu.my (W.Y.C.); YIP.KHARYEE@student.imu.edu.my (K.Y.Y.)

**Keywords:** ocular drug delivery, mucoadhesive, in situ ophthalmic gel, thermo-responsive, pH-responsive, ion-responsive, multi-stimuli-responsive, novel approaches, safety

## Abstract

Eyesight is one of the most well-deserved blessings, amid all the five senses in the human body. It captures the raw signals from the outside world to create detailed visual images, granting the ability to witness and gain knowledge about the world. Eyes are exposed directly to the external environment; they are susceptible to the vicissitudes of diseases. The World Health Organization has predicted that the number of individuals affected by eye diseases will rise enormously in the next decades. However, the physical barriers of the eyes and the problems associated with conventional ocular formulations are significant challenges in ophthalmic drug development. This has generated the demand for a sustained ocular drug delivery system, which serves to deliver effective drug concentration at a reduced frequency for consistent therapeutic effect and better patient treatment adherence. Recent advancement in pharmaceutical dosage design has demonstrated that a stimuli-responsive in situ gel system exhibits the favorable characteristics for providing sustained ocular drug delivery and enhanced ocular bioavailability. Stimuli-responsive in situ gels undergo a phase transition (solution–gelation) in response to the ocular environmental temperature, pH, and ions. These stimuli transform the formulation into a gel at the cul de sac to overcome the shortcomings of conventional eye drops, such as rapid nasolacrimal drainage and short contact time with the ocular surface This review highlights the recent successful research outcomes of stimuli-responsive in situ gelling systems in treating in vivo models with glaucoma and various ocular infections. Additionally, it also presents the mechanism, recent development, and safety considerations of stimuli-sensitive in situ gel as the potential sustained ocular delivery system for treating common eye disorders.

## 1. Introduction

Worldwide, eye disorders and consequential visual impairment are recognized as the nation’s absolute menace, compromising physical and mental health [1]. To date, 90% of the commercialized ophthalmic products consist of eye drops for treating many ocular diseases, such as infection, glaucoma, cataract, inflammation, dry eye, and allergy [2]. The topical administration of ocular drugs remains the most widely accepted treatment route due to hassle-free and least invasiveness [2,3]. Nevertheless, the complex ophthalmic environment equipped with static, dynamic, and metabolic barriers makes topical drug delivery rather challenging [4,5]. Static barriers are the physical stumbling blocks of biological membranes that drugs must pass through to exert a pharmacological effect [2,6]. The biological membranes have distinguishing thickness, hydrophilicity, hydrophobicity, and collagen content that hamper drug passage. Dynamic barriers consist of nasolacrimal and lymphatic drainage and conjunctival blood flow [2,4]. Metabolic barriers involve efflux pumps or enzymes that may deactivate drugs. Altogether, these factors lead to accelerated precorneal drug clearance, low ocular contact time, and limited drug ocular bioavailability (<5%) [7]. Having to compensate for the drug loss, high drug concentration, and repeated administration are required to reach the targeted therapeutic concentration, which sometimes, undesirably, results in adverse effects and poor patient adherence [7]. Thus, it has become the impetus for pharmaceutical scientists to continuously improvise and design innovative, efficacious and cost-effective ocular dosage forms. The ultimate strategies to improve ocular bioavailability upon topical instillation are to increase precorneal retention time and enhance drug permeability across the ocular barriers [8].

In recent years, the development of stimuli-responsive in situ gel has received substantial attention as smart ocular delivery systems. Stimuli-responsive in situ gels are formulated using environment-sensitive, biocompatible, non-irritant polymers that undergo phase transition (solution–gelation) in response to the ocular environmental stimuli temperature, pH, and ions [7]. Formulation scientists can manipulate the concentration of stimuli-responsive in situ gelling polymers to control the rate and extent of gel formation, ocular residence time, and drug release rate, without requiring additional organic solvents or co-polymerization agents to trigger gel formation [3,4]. The in situ gelling system is unique as it can be administered as a liquid into the eye, as easy and non-invasive as the eye drop, thereby delivering a precise amount of drug. When in situ formulation transforms into the gel at the cul de sac of the eye, they overcome the shortcomings of eye drops such as rapid nasolacrimal drainage and short contact time with the ocular surface [2,6]. Some of the in situ gelling polymers are mucoadhesive, forming attractive interactions with the corneal mucous layer [3]. The use of mucoadhesive in situ polymers can significantly lengthen the ocular residence time to achieve sustained drug delivery. While comparing an in situ gel system with ointment, the latter often causes a stinging, burning sensation and vision blurriness, which compromises visibility [3,4]. Meanwhile, suspensions are usually associated with drug non-uniformity and unpredictable ocular bioavailability compared with in situ gels [2,4]. This review presents the mechanism, recent development, and safety considerations of stimuli sensitive in situ gel as the potential sustained ocular delivery system for treating common eye disorders.

## 2. Mucoadhesive Polymeric Approach

**Figure 1:**
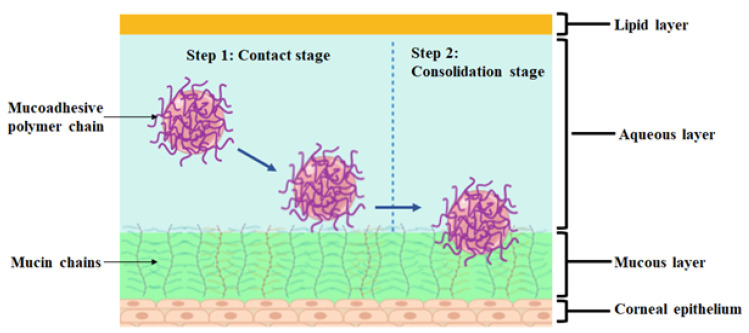
Mucoadhesion involves two steps: a contact stage followed by a consolidation stage. In the contact stage (Step 1), wetting and swelling occur in the mucoadhesive formulation when the polymer spreads over the mucus membrane to develop a deep contact with the mucus layer [13,14]. Subsequently, the consolidation stage (Step 2) involves physicochemical interactions that establish and consolidate the adhesive interaction [15]. In this stage, the mucin chains entangle with the mucoadhesive polymer chains, resulting in mechanical bond formation [16,17]. Then, moisture’s presence promotes the formation of chemical bonds such as hydrogen bonds, covalent bonds, and weak van Der Waals forces that strengthen the system [13,16].

As discussed above, the physical barriers and the problems associated with conventional ocular formulations are the significant challenges in ophthalmic drug development. The mucoadhesive polymeric approach has gained much importance in offering several advantages to confront the challenges. This approach has been used for drug delivery at different body sites such as buccal, gastrointestinal, nasal, rectal, vaginal, and ocular, as it offers several advantages [9,10]. It is described as the interaction resulting from attractive forces between synthetic or natural polymers with the mucous membrane [11,12]. Figure 1 is an illustrative diagram showing the mechanism of mucoadhesion.

Mucoadhesive polymers have the advantage of lengthening the residence time at the application site to achieve sustained drug delivery. In this context, Moustafa et al. developed a gel composed of fluconazole and Carbopol gel-core tested on rabbits’ eyes. The study revealed that the incorporation of mucoadhesive Carbopol lengthened the ocular retention time for more than 18 h compared to the fluconazole suspension and conventional liposomes [18]. The study done by Abd El-Bary et al. is agreement with the previous study. The drug release profile revealed that Carbopol significantly retarded the drug release of levofloxacin ocular mini-tablets for up to 24 h attributed to its excellent mucoadhesive properties. This is because the gelatinous layer formed on the mini tablets’ surface upon wetting, which restrains the drug release from the formulation [19]. Evidently, the incorporation of mucoadhesive polymers increases ocular residence and provides sustained release action upon contact with the mucous membrane. Moreover, the mucoadhesive delivery system enables the drug release at targeted sites, hence bypassing first-pass degradation [16]. Thus, this system promotes local absorption due to the rich blood supply of mucosa, provides a localized effect, and subsequently improves ocular bioavailability along with therapeutic efficacy [5]. Morsi et al. synthesized acetazolamide nanoemulsion-based in situ gel using xanthan gum, hydroxypropyl methylcellulose (HPMC), or Carbopol in the blend with gellan gum for glaucoma therapy. The AUC of gellan gum/xanthan gum formulation is 2.29-fold and 2.37-fold greater than Azopt^®^ eye drops and Cidamex^®^ oral tablets. Significantly, the formulation prolonged the intraocular pressure (IOP) lowering effect in glaucomatous rabbits’ eyes. It could be deduced that the therapeutic efficacy was enhanced by virtue of the mucoadhesive properties of xanthan gum [20]. On top of that, the dosing frequency can be reduced, which in turn minimizes the possible side effects, resulting in better patient acceptance and compliance [12,21]. Park and co-workers investigated potential vehicles for dorzolamide delivery by comparing the marketed dorzolamide eye drop Trusopt^®^ against different formulations, among them mucoadhesive (MUCO_NM) ocular tablets [22]. The researchers found out that MUCO_NM demonstrated the highest therapeutic efficacy compared to other formulations. Remarkably, a 4-fold higher dose of Trusopt^®^ is required compared to MUCO_NM tablets, which required a single administration to achieve a comparable therapeutic effect. The outcome suggested that the mucoadhesive properties of the MUCO_NM have enhanced treatment outcomes with a reduced frequency of dorzolamide topical administration [22].

## 3. Smart Ocular Delivery Using Stimuli-Responsive In Situ Gel Platform

### 3.1. Thermo-Responsive In Situ Ocular Gelling Systems

Thermo-responsive in situ ocular gelling systems are the most extensively explored stimuli-responsive in situ gelling system. This polymeric system undergoes phase transition (solution → gel) in response to temperature change due to the increase in hydrophobicity [23]. It is recommended that an ideal thermosensitive in situ gel should undergo sol–gel transition upon contact to the eye physiological temperature to prevent the ophthalmic in situ gel from being stored in the fridge, which may cause eye irritation due to low temperature [6]. Innumerable thermo-responsive in situ approaches have been employed in the treatment of various ocular diseases such as glaucoma, bacterial infections, fungal infections, viral infections, and others. The commonly used polymers can be broadly classified into natural polymers (cellulose derivatives) and synthetic polymers (including poloxamers and poly(N-isopropylacrylamide) or PNIPAAM). Approaches to improve the ocular delivery of therapeutics using the thermo-responsive platform have been summarized in Table 1.

#### 3.1.1. Recent Research on the Applications of Thermo-Responsive In Situ Ocular Gelling Systems

##### Ocular Hypertension (Glaucoma)

Glaucoma refers to a condition associated with elevated IOP, which can cause damage to the optic nerves known as optic neuropathies. The irreversible damage to the optic nerves can eventually lead to vision loss if left untreated [40]. The topical agents for glaucoma therapy include beta-blockers, prostaglandin analogues, alpha agonists, and carbonic anhydrase inhibitors [40]. There are demerits associated with the conventional anti-glaucoma eye drops such as limited drug absorption, poor bioavailability, poor patient compliance, undesirable systemic side effects, and inaccurate dosing [40,41]. Several researchers have successfully developed in situ gelling systems to accomplish effective drug delivery for glaucoma therapy. In this context, Khattab et al. fabricated a thermo-responsive in situ gel of latanoprost for glaucoma by utilizing Pluronic F-127, Pluronic F-68, HPMC E5, and HPMC E50. The gelation temperature of the optimized formulation was found at 34.3 °C. The in vitro drug release studies suggested prolonged release with the increase in the concentration of poloxamer and HPMC due to higher viscosity. The mucoadhesive properties of HPMC also play a role in retarding drug release due to their ability to compress the aqueous channel of the poloxamer micelles [24]. The kinetic release data depicted the formulation followed non-Fickian diffusion release mechanism and fitted the Higuchi model. Furthermore, an ex vivo permeation study in cow cornea displayed improvement in permeation due to the presence of Pluronic F-127, as it can interfere with the lipid membrane results in leaking of protein and lipid, and hence enrich the transcellular transport. Subsequently, the optimized formulation was evaluated for anti-glaucomatous efficacy in the rabbit glaucoma model. The formula showed superior efficacy compared to Ioprost ^®^ with a 2.9-fold higher AUC value without any sign of ocular tissue damage and irritation. The outcome suggested that the formulation possessed a lengthened residence time, which enhanced both ocular bioavailability and therapeutic outcome [24].

**Figure 2:**
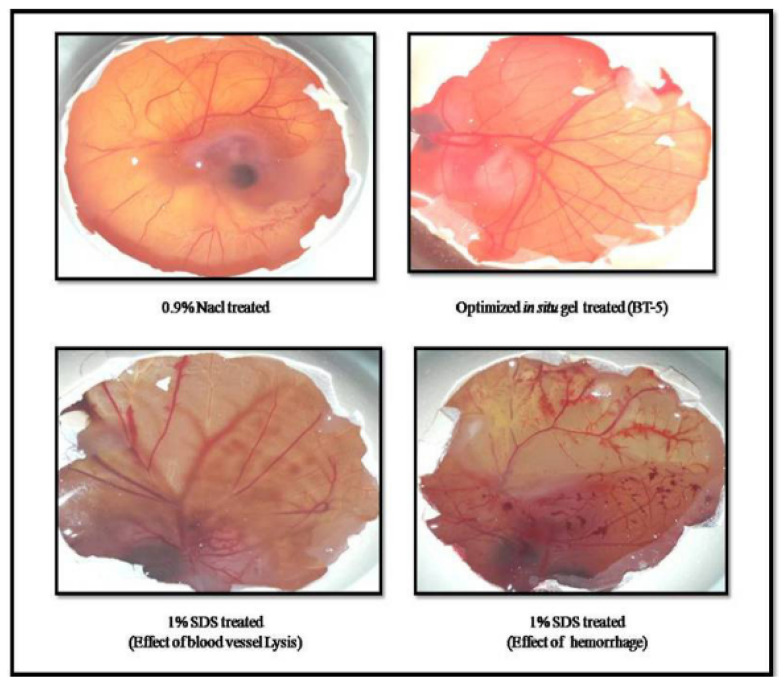
HET-CAM test for optimized formulation. Each of the eggs is treated with 0.9% NaCl (negative control), 1% sodium dodecyl sulfate (SDS) (positive control), and optimized formulation, respectively. The mean irritation score obtained for the negative control, positive control, and the optimized formulation was 0.04 (no irritation), 14.07 (severe irritation), and 0.05 (no irritation), respectively [25].

A similar approach has also been used by another group of researchers to improve the ocular delivery of bimatoprost. The in situ gel was prepared by using poloxamer 188, poloxamer 407, and HPMC K4M. The study demonstrated that the gelation temperature of the optimum formula was 37.5 °C. The formulation exhibited rapid gelation and remained for 10 h. The increment in gelling capacity can be justified by the fact that more micelles were formed as the concentration of poloxamer was higher. In this study, the viscosity was increased when the concentration of HPMC K4M used was higher, as this polymer served as a viscosity enhancer. The optimized formulation released 80.37–86.63% of the drug for 10 h and fitted the Higuchi model. Ex vivo drug permeation study using goat cornea showed that 67.45% of the drug was permeated up to 12 h. Hence, the findings confirmed the longer time for drug release, and better permeation can be achieved by incorporating thermo-responsive in situ gel. However, the researchers had not compared the results with a marketed or conventional product. Moreover, the researcher employed the HET-CAM method to evaluate ocular irritation for optimized formulation, as shown in Figure 2 [25].

Betaxolol hydrochloride (BH) is a beta-blocker, which has been used in the treatment of glaucoma. The commercially available BH eye drop known as Betoptic S^®^ is in resin suspension form. In spite of the fact that resin suspension offers a sustained release profile, it requires an ionizable drug that can form a complex with the resin [26,40]. In this regard, Wei et al. strived to develop a formulation by employing poloxamer 407 and an MC-based thermosensitive in situ gelling system to tackle the drug selectivity problem and improve the ocular delivery of BH [26]. The gelation temperature of the poloxamer-based gel was found at 29.16 ± 0.21 °C, and it was reduced when a higher concentration of poloxamer 407 was used. This is because the number of micelles increases; thereby, less energy is needed for micellar crystallization. The addition of HPMC can achieve the desired gelation temperature while decreasing the concentration of poloxamer, which can avoid irritation potential. Whereas for MC-based in situ gel, the higher concentration of MC further decreases the gelation temperature due to hydrophobic interaction [42]. However, the gelation temperature is still not acceptable (≈45 °C) when the concentration of MC was as high as 5%. The addition of PEG resolved this issue, as it is a proton acceptor that strengthens the hydrophobic interaction of MC, resulting in a gelation temperature of 34 °C. The in vitro release profile showed that both gels shared a similar sustained-release profile. An in vivo tissue distribution study was performed in rabbits, and the results pointed out that both formulations exhibit similar drug concentrations in corneal, iris-ciliary, and aqueous humor. The AUC and mean retention time (MRT) of both in situ formulations were 2-fold higher than the marketed suspension eye drops [26].

On top of that, timolol maleate (TM) is another potent beta-blocker for glaucoma therapy, but its therapeutic effect is often limited by poor corneal permeability and short drug retention time [27]. Zeng et al. attempted to address the issues by implementing a thermo-sensitive in situ gel technique. The phase transition of the thermosensitive gel occurred at 32 °C, which is acceptable for ophthalmic preparations. In vivo pharmacokinetics findings in the rabbits model demonstrated that the MRT of TM in situ gel was 1.6-fold greater than TM eye drops. The AUC of the optimized formulation was also higher than TM eye drops, from which it could be deduced that thermosensitive in situ gel had better bioavailability and treatment efficacy. This can be reinforced by the in vivo pharmacodynamics study in glaucomatous rabbits’ model where it was demonstrated that the TM-loaded in situ gel possessed a more significant IOP-lowering effect compared to TM eye drops [27]. Notably, the in situ gelling system plays a splendid role in increasing desired ocular effects, and hence, it can be a good candidate for the ophthalmic drug delivery of glaucoma therapeutic agents.

##### Ocular Bacterial Infections

Ocular bacterial infections are also another significant cause of blindness. Conjunctivitis is often caused by bacteria; it is an inflammatory condition where the clinical manifestations include irritation, tearing, mucopurulent discharge, and even impaired vision [43]. The common causal bacteria include *Staphylococcus aureus*, *Streptococcus pneumoniae*, *Haemophilus influenzae*, etc. [43,44]. Topical antibiotics are the mainstay of treatment options for bacterial conjunctivitis. Koland and Prabhu produced and examined a thermo-responsive in situ ophthalmic gel of ofloxacin with the use of poloxamer 407 and mucoadhesive polymers such as HPMC and polyvinyl alcohol (PVA) to lengthen the corneal retention time and drug release [28]. The prepared formulations demonstrated sustained drug release for 9 h where the drug release was slower as the concentration of poloxamer and mucoadhesive polymers was higher due to increased viscosity. It was reported that the incorporation of HPMC decreased the gelation temperature while PVA had no significant effect on gelation temperature and drug release. In vitro release data represented that all formulations obeyed first-order drug release and Higuchi’s Matrix model. All in situ formulations are effective in antimicrobial, but the zone of inhibition (ZOI) was smaller compared to the marketed formulation (0.3% *w*/*v* ofloxacin eye drops) [28].

On the other hand, Levofloxacin is an L-isomer of ofloxacin that was found to have higher activity compared to ofloxacin [45]. Saher and colleagues attempted to harness thermosensitive in situ gel composed of levofloxacin hemihydrate, Pluronic F-127, Pluronic F-68, and sodium perborate monohydrate as a disappearing preservative [29]. The optimized formulation underwent phase transition at 32.75 ± 0.35 °C. Similar to previous studies mentioned, the higher concentration of Pluronic F-127 would result in a lower gelation temperature. In contrast, the addition of Pluronic F-68 increased the gelation temperature [29] by perturbing the hydration sphere, give rise to a lower order of water molecules around the hydrophobic polyoxypropylene (PPO), which decreased the hydrophobic interaction between micelles, and thus, a higher temperature would be needed to strengthen the interaction in gelation [46]. An in vitro drug release study revealed that the optimized formulation exhibited prolonged drug release up to 12 h. In addition, the release data conformed the Higuchi model and represented a non-Fickian release. In vivo results demonstrated that the developed formulation had a longer retention time (12 h) in rabbits’ eye fluid compared to Levoxin^®^ eye drops (4 h) without visual signs of irritation. In short, the study encouraged the use of thermosensitive polymers to attain the sustained release of therapeutic agents at target ocular sites [29].

Sparfloxacin is a third-generation fluoroquinolone, which is another effective topical medication for bacterial conjunctivitis. The dissolution rate of sparfloxacin is often limited by its poor solubility; ergo, Sawant et al. exploited emulsomes as a drug delivery vehicle and thermosensitive in situ gel to improve drug permeability, extend drug release, and increase ocular retention time [30]. The optimized emulsomes and sparfloxacin were dispersed in the poloxamer-based in situ gel, which was composed of Pluronic F-127 and Pluronic F-68. The in vitro release studies showed that the drug release of the optimized formulation was significantly lengthened for more than 12 h, which was slower than pure drug dispersed in the gel base attributed to the sustained action of emulsomes. An ex vivo goat cornea permeation study reported that the drug release was lesser than the in vitro dialysis membrane study because of the lipophilic–hydrophilic barrier present in the cornea while the dialysis membrane is a mechanical barrier. The induced conjunctivitis rabbits’ model was treated with the optimized formula, the symptoms were reduced within 4 to 5 days, which was faster than the pure drug solution without any sign of ocular irritation [30].

**Figure 3:**
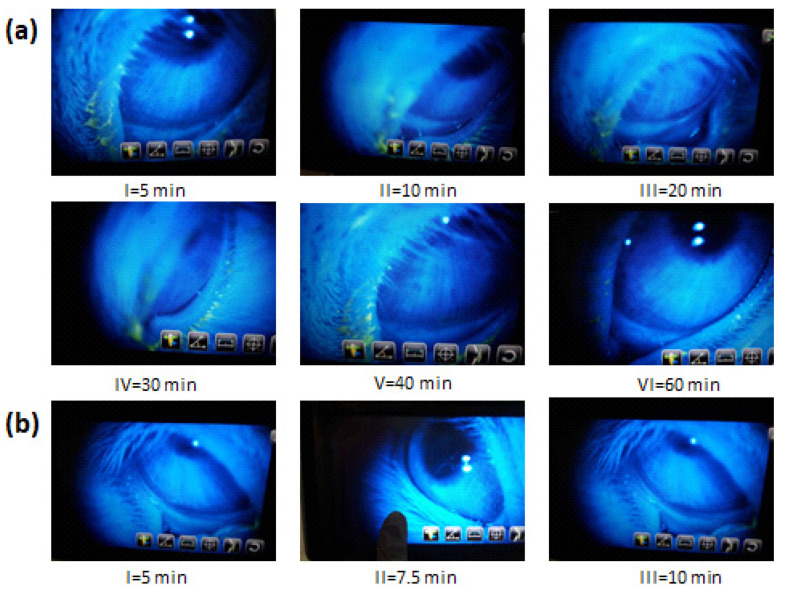
(**a**) In vivo fluorescein tracking study/elimination study. Before administration, fluorescein sodium salt was incorporated into the formulations to track the presence of eye drops. One drop of marketed solution eye drop of ciprofloxacin was administered in the lower conjunctival sac. An auto-refractometer visualized and showed that the elimination of the marketed eye drop was within 10 min [31]. (**b**) In vivo fluorescein tracking study/elimination study. Similarly, fluorescein sodium salt was incorporated into the formulations to track the presence of gel. One drop of in situ gel was administered in the lower conjunctival sac. An auto-refractometer visualized and showed that the elimination of the marketed eye drop was within 60 min [31].

Concurrently, researchers have also developed thermosensitive in situ gel for the treatment of bacterial keratitis. In this regard, ophthalmic ciprofloxacin in situ gel was developed using poloxamer 407 in combination with HPMC as a viscosity enhancer. The formulation underwent rapid gelation at 30 ± 0.01 °C and remained for an extended period. An in vitro release study revealed that the release of optimized in situ gel was prolonged for 8 h, whereas the marketed eye drops were released immediately within 10 min. Furthermore, in vivo elimination studies displayed the elimination of solution drop and optimized in situ gel within 10 min and 60 min, respectively (Figure 3a,b) [31].

A comparative study on the impact of pre-formed gels (MC and CMC) and Pluronic F-127 in situ gel on the ocular drug delivery of ciprofloxacin hydrochloride has previously been reported. The phase transition temperature of the Pluronic in situ gel was 35 °C. Although MC-based gel was a pre-formed gel, it has thermal–gelation properties as its viscosity was increased with higher temperature. The appearance of all gels was transparent except for CMC-based gels, which may be caused by the precipitation of ciprofloxacin hydrochloride with the sodium salt presented in CMC-based gels. The researchers have investigated that all gel formulations exhibited sustained release compared to ciprofloxacin solution in vitro. In addition, it was found that the higher concentration of Pluronic F-127 leads to slower release, where similar trends were observed for CMC and MC-based gel attributed to the greater viscosity. Higuchi and Korsmeyer–Peppas were the best models to explain the release profile of the formulation, and the release mechanism followed the non-Fickian release. An in vivo study in bacterial keratitis rabbits’ model (*Staphylococcus aureus* infected) displayed faster healing for both pre-formed gels and Pluronic in situ gel in less than 5 days with twice-daily dosing compared with ciprofloxacin solution, which required 7 days to heal with four times daily dosing [32]. Markedly, the drug release, resulting in less frequent dosing without diminishing the therapeutic effect. Although the pre-formed gel and the in situ gel produced similar outcomes, the in situ gel provides consistent administration of therapeutic agents unlike pre-formed gel [20,31]. Moreover, the study has reported that Pluronic F-127 in situ gel causes significant stromal edema compared to MC and CMC pre-formed gel [32]. The histological examination of this study will be further discussed in the safety profile section.

##### Ocular Fungal Infections

Ocular infectious keratitis can also be caused by fungal species where the common causal species are *Aspergillus*, *Fusarium*, *Candida*, *Curvularia*, and *Penicillium*. The prognosis of fungal keratitis is poor owing to lack of effective treatment, and fungal pathogenesis is different from other pathogens [47]. Notwithstanding the fact that natamycin is used as the first-line treatment for fungal keratitis, other antifungals such as voriconazole, ketoconazole, and fluconazole, etc. can also be used [48]. Voriconazole possesses low solubility in the gastric fluid, which may affect the bioavailability as well as the therapeutic outcome. Hence, Üstündağ Okur et al. developed voriconazole-loaded thermosensitive in situ ocular gel utilizing poloxamer 188, poloxamer 407, and/or CMC to extend the retention time and enhance the bioavailability of voriconazole [33]. The selected formulation underwent gelation at 34.13 ± 0.32 °C. The in vitro release profile revealed that sustained release was achieved where the release can be further retarded with higher polymer concentration. Plus, the addition of CMC further retarded the drug release compared to poloxamer alone. Ex vivo permeation and penetration studies using goat corneas showed that the formulation had better permeation than another formulation because the presence of CMC prolonged the release. Contrarily, the formula with the addition of 0.3% CMC demonstrated better penetration compared to the one without CMC due to the mucoadhesive effect of CMC. However, the formula without the addition of CMC was further evaluated in vivo because the researchers reported that the slow release rate (50% after 24 h) of the formulation with CMC limits their further studies. In vivo studies compared 50 μL optimized in situ gel and voriconazole solution in New Zealand White (NZW) rabbits’ eyes where the results showed the drug concentration in tear fluid was higher for in situ gel without causing ocular damage [33]. More recently, in another work by another group of researchers, voriconazole-loaded thermosensitive in situ gels were prepared based on poloxamer 188, 407, and 388. The optimized in situ gel underwent gelation within 1.233 ± 0.058 s at 32.200 ± 0.265 °C. Particularly, the addition of poloxamer 188 lengthened the gelation time and increased the gelling capacity. Similar in vitro release study results were observed in which the formulations showed sustained release up to 24 h [34]. Nonetheless, in vivo studies for ocular irritation and retention, and ex vivo permeation studies had not been conducted in this study compared to the previous study. In addition, both studies [33,34] had not conducted in vivo antimicrobial efficacy to show the potential clinical use of the novel formulations.

On the other hand, Zhu et al. fabricated ketoconazole-loaded thermosensitive in situ gel with the use of PNIPAAM-hyaluronic acid (HA). The formulation was converted into a gel at 33 °C. An in vitro release study illustrated that a slower rate of release was observed in ketoconazole in situ gel when compared to free ketoconazole. During the first 2 h, 30% of ketoconazole was released from in situ gel, while 95% of ketoconazole was released from the free drug group. The analysis of release kinetics suggested that the release was best expressed by the Higuchi model. Apart from this, the results obtained from in vivo studies pointed out that ketoconazole-loaded PNIPAAM-HA in situ gel exhibited the highest cure rate with 91.7% compared to commercial ketoconazole eye drops and placebo with the cure rate of 66.7% and 0%, respectively. The ocular irritation test was performed in NZW rabbits, and the irritation score indicated that this formulation did not cause irritation and can be used safely in ophthalmic preparations. Seemingly, the increased residence time and controlled drug release were obtained from this novel in situ ocular formulation [35].

##### Ocular Viral Infections

Herpes Simplex (HSK) is one of the important causes of blindness worldwide. As discussed, the therapeutic outcome and patient compliance are affected due to some of the limitations of the available formulations. Mahboobian and colleagues designed acyclovir (ACV)-loaded thermo-responsive in situ gel nanoemulsions utilizing Triacetin, Transcutol^®^ P, poloxamer 407, and poloxamer 188 to overthrow the shortcomings of conventional formulations. In this study, the gelation temperature of the optimized formulation was within the acceptable range (30.9 °C). According to the in vitro release results, the optimized formulations displayed a sustained release manner. There was only 55–60% of ACV released compared to ACV solution (control), which approximately released the total amount of drug after 30 min. The authors suggested that the entrapped drug in nanoemulsion slowed down the drug release from the oil phase to the aqueous phase. Ex vivo results showed that the permeation of optimized formulation through the bovine corneal membrane was 2.83-fold higher than that of the ACV solution, which can be explained by the presence of poloxamer 407 leading to the disruption of mucus and reduction of its integrity. In addition, the nano-sized particles were easier to be uptaken by the corneal epithelium cells, and the lipophilic structure of nanoemulsion enhanced the ACV permeation. An ocular irritation test was performed in three rabbits, and minimal conjunctival redness was observed in one rabbit. However, the Draize score of this formulation did not exceed 0.33, and the redness gradually decreased and disappeared at 2 h post-instillation. A similar result was replicated in the HET-CAM test where hyperemia was observed in one out of three chorioallantoic membranes, and the irritation mean scores revealed that the optimized formulation was practically non-irritant [36]. Remarkably, the combination of thermosensitive polymers and nanoemulsion technology can further enhance the permeation of poor solubility of ACV and increase its ocular bioavailability, which may result in better treatment outcomes.

##### Other Ocular Diseases

The use of thermosensitive in situ gelling systems in the treatment of other diseases including corneal neovascularization, ocular allergy, and cataract has been studied [37,38,49,50,51,52]. Corneal neovascularization is related to the invasion of new blood vessels into the cornea from the limbus because of an imbalance between angiogenic and antiangiogenic factors, which can be caused by ocular injury and diseases [52]. Recently, a nintedanib eye drop has been shown to have potential in reducing the development of choroidal neovascularization in mice, but it may not provide a long-lasting effect, owing to rapid elimination [49]. Henceforth, Gong et al. prepared a nintedanib thermosensitive hydrogel (NTH) utilizing poloxamer 407 tested on the alkali burn rat model [37]. The gelation temperature was found to be 37 °C. In vivo studies showed that the corneal neovascularization (CNV) area in the chloramphenicol-treated group (model group) reached the maximum at day 14. In contrast, the 0.2% NTH-treated group showed a reduced CNV area at each time point (day 3, 7, and 14), indicating that the growth rate of neovascularization in the NTH-treated group was slower. The inhibition effect of NTH was significantly as effective as 1% dexamethasone ointment. vascular endothelial growth factor VEGFR-2 plays a role in promoting intraocular neovascularization, while CD31 represents the relative levels of vascular endothelial cells in the cornea. The authors carried out immunofluorescence staining and found out that the expression of VEGFR-2 and CD31 was downregulated in the NTH-treated group [37]. Considerably, thermosensitive hydrogel improved the delivery of nintedanib and effectively reduced neovascularization.

Ocular allergy is another common ocular disorder; the symptoms such as itchiness can negatively impact patients’ quality of life and productivity, leading to economic burden. Topical therapeutic agents have been used to alleviate symptoms by suppressing inflammatory responses [38,50]. Recently, Üstünda and co-workers developed and evaluated a thermosensitive dispersion of tetrahydrozoline (THZ)-loaded in situ gel (composed of poloxamer 188 and poloxamer 407) for ocular allergy. The gelation of the optimized formulation occurred at 31.08 ± 0.34 °C, and its pH value was 7.010 ± 0.017. An in vitro release study was carried out illustrating prolonged release in which 88.745 ± 8.275% of THZ was released from optimized in situ gel up to 24 h. Furthermore, 2.035 ± 0.062% of THZ permeated through the goat cornea, whereas 0.582 ± 0.035% of THZ penetrated the corneal tissue. Nevertheless, ex vivo studies did not compare the optimized formulation with the marketed product of THZ to show it has better permeation and penetration. An in vivo study was performed to determine the concentration of THZ in rabbits’ tear samples after the instillation of 50 μL of developed formulation and marketed product of THZ. Initially, the concentration of THZ in tear samples for the commercial product and optimized formulation was found to be 3.4 μg/mL and 9.47 μg/mL, respectively. At 6 h, THZ was not detectable for the marketed product, while 1.029 μg/mL of THZ was detected for the developed formulation. In addition, the ocular damage was not observed in both the optimized formulation and commercial product [38]. In short, thermosensitive in situ gel is a favorable approach to increase the ocular retention time and drug release, simultaneously improving the bioavailability and therapeutic outcome of ocular allergy.

**Figure 4:**
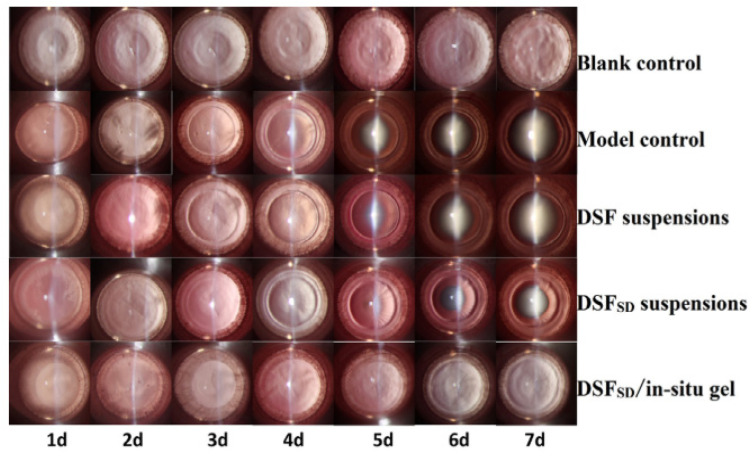
The anti-cataract effect of the blank group, model group, DSF suspensions group, DSF_SD_ suspensions group, and DSF_SD_/in situ gel group were evaluated throughout the 7 days of administration. (Blank control): the lenses were clear; (Model control): the opacity was observed and developed cataracts; (DSF suspension): the cataract was developed faster on day 4 and day 5 compared to the model group; (DSF_SD_ suspension): the cataract was developed but not as severe as the DSF suspension group; (DSF_SD_/in situ gel): the lenses were clear, and no opacity was detected throughout 7 days [39].

A cataract is a sight-threatening condition due to the opacification of the lens. Cataract surgery is the most common intervention for cataracts. However, it is an invasive method that results in several complications including cystoid macular edema, corneal edema, retinal detachment, intraocular hemorrhage, and endophthalmitis [51]. Disulfiram (DSF) is a prodrug of diethyldithiocarbamate that had been reported to be effective in inhibiting cataract development, but its poor aqueous solubility limits its efficacy [39,53]. Zhang et al. attempt to resolve this issue by employing a solid dispersion technique and a thermosensitive in situ gelling system. Initially, the researchers prepared DSF solid dispersion (DSF_SD_) by the hot-melt method. The prepared DSF_SD_ was further incorporated in poloxamer 407 and poloxamer 188-based in situ gel. The formulation with the ratio of 1:5 (DSF to poloxamer 188) showed the highest apparent permeability coefficient (P_app_), which was 2.31-fold higher than DSF eye drops. The fluorescence precorneal retention study was carried out using rhodamine B, as DSF has no fluorescence absorption. The findings illustrated that in situ gel successfully prolonged the retention of fluorescence for 30 min, while the suspension was rapidly cleared out by lachrymal fluid. An in vivo study evaluated the cataract effect among normal rat pups (blank control) and selenium-induced cataract rat pups (model control). The other three groups of selenium-induced cataract rat pups were treated with DSF suspensions, DSF_SD_ suspension, and DSF_SD_/in situ gel, respectively. At day 7, the lenses were clear in both the blank group and DSF_SD_/in situ gel group, whereas the opacity was observed in the other three groups. However, DSF_SD_ suspension was not as severe as the model group and DSF suspensions group (Figure 4) [39]. As a result, the study proposed that thermosensitive in situ gel offers effective ocular delivery by extending the drug retention time and improving treatment outcome for cataract.

One of the leading causes of blindness in diabetic patients is diabetic retinopathy (DR). To treat DR, a temperature-sensitive gel embedded with curcumin-loaded albumin nanoparticles was developed. The transition temperature of the optimized formulation was above 34.2 °C. The gel showed sustained release properties and the insertion of nanoparticles did not affect the gel structure. Additionally, an in vivo eye irritation test on a rabbit eye showed the formulation as safe for administration without any eye irritation and also enhanced the bioavailability of curcumin in aqueous humor [54]. Similarly, Deguchi et al. designed disulfiram (DIS) nanoparticles embedded in situ gel based on methylcellulose (MC) for the treatment of DR. The formulation was stable, as not aggregation or participation was reported in 1 month and gelled at 37 °C. Prolonged retinal residence time was observed after the instillation of gel. These studies have shown the effectiveness of in situ gels in the treatment of retinopathy [55].

### 3.2. pH-Responsive In Situ Gelling Systems

The other type of stimuli-responsive approach is the external pH environment. Generally, these polymers are polyelectrolytes in which there are repeating units of acidic or basic groups. In weak acidic group polymers (polyacids), it displays swelling properties with increasing environmental pH and vice versa for weak basic group polymers (polybases). The protonation of these polymers results in electrostatic repulsion and the build-up of a network-like matrix, thereby causing a size expansion and swelling appearance [6]. In formulating ocular drug delivery, these polymers are expected to gel upon contacting the neutral pH of the ocular surface. Various approaches to formulate the pH-responsive gelling system for improved ocular delivery of therapeutics have been summarized in Table 2.

#### 3.2.1. Recent Researches on the Application of pH-Responsive In Situ Ocular Gelling Systems

##### Ocular Hypertension (Glaucoma)

Glaucoma is an ocular disease involving optic nerve damage, accompanied by raised IOP leading to vision loss.

Gupta and colleagues previously developed timolol pH-responsive in situ gel by employing two pH-sensitive polymers, carbomer and chitosan [56]. Chitosan played a role as a viscosity enhancer and lowered the concentration needed for carbomer to form a stiff gel [56,77]. Comparing the optimized in situ gel with liposomes and marketed drops (Glucomol^®^) disclosed that the developed in situ gel formulation could release up to 24 h steadily, obeying Fickian diffusion comparable with liposomes formulation. Despite the sustained release of the liposome, it was notifiable that the liposome did not remain intact with the corneal surface. An in vivo study performed for determining the efficacy of timolol in the respective formulation revealed a 1.2-fold increment in liposome formulation while there was a 2.5-fold increment in the in situ gel compared with Glucomol^®^. The peak IOP-lowering effect was significantly prolonged up to 7 h in the in situ gel, while others started to decline after 3 h. The authors also reported low systemic absorption in the in situ gel and liposome formulation, whereas Glucomol^®^ showed a spike in serum drug concentration after 10 min instilled in the eyes. This is mainly due to the rapid nasolacrimal drainage of the ophthalmic solution. Although the liposome formulation did not provide extended precorneal contact time, formulating the liposome together with in situ gel can be carried out instead of individually comparing to investigate whether it provides a possible synergistic release profile [56].

Intriguing, Dubey et al. blended two active ingredients, timolol and brimonidine, concurrently in the in situ gel system utilizing Carbopol 934P [57]. Both incorporated drugs were found to be compatible, and the formulation was further explored. The in situ gel formulation provided was sustained up to 8 h with a zero-order release rate in agreement with the previous study. The formulation was tested on white rabbit eyes that exhibited normal iris, conjunctival blood vessels, absence of ocular swelling, and normal blinking reflex. The highlight of this study was the in vivo IOP lowering activity that was performed on rabbit models. An excellent IOP-lowering effect was observed in the in situ gel formulation for up to 12 h while marketed ophthalmic drops were able to delay up to 5 h only.

Furthermore, several studies that developed brimonidine by a pH-responsive strategy also supplement the use of an in situ gel system in controlling glaucoma condition. Pang and co-authors developed various concentrations of brimonidine (0.05%, 0.1%, and 0.2%) in the in situ gelling systems and compared the in vivo studies as well as biocompatibility with 0.2% brimonidine ophthalmic eye drops [58]. An extensive in vivo study was carried out on NZW rabbits to determine the pharmacodynamic and pharmacokinetic of brimonidine. The outcome revealed that all in situ gel formulation concentrations reduced the IOP level with excellent AUC plotted in the IOP-lowering time curve compared to 0.2% brimonidine drops. The lowest concentration (0.05% brimonidine) in situ gel showed 1.2-fold greater AUC compared to 0.2% brimonidine drops. Other concentrations were 1.6-fold and 2.4-fold better corresponded to 0.1% and 0.2% brimonidine in situ gel. This suggested that despite having lower brimonidine strength, the in situ gel maximized the therapeutic efficacy of the brimonidine, as the formulation could retain on the corneal, permitting constant release and drug penetration to the corneal layers. This statement was further supported by conducting precorneal residence time on the corneal surface and conjunctival sac of the rabbits with a fluorescence dye. The in situ gel formulation appeared to be 3 h longer than ophthalmic drops. Moreover, the pharmacokinetics study showed a spike in plasma concentration in ophthalmic drops while in situ gel with all concentrations remained low, suggesting that it could lower the systemic absorption. Hence, possible systemic side effects such as dry mouth, drowsiness, and cardiorespiratory symptoms, resulting from a substantial amount of drug drainage via nasolacrimal ducts could be prevented [78].

Similarly, Barse et al. also developed brimonidine and explored the release kinetics and permeability testing [59]. Employing the same polymer Carbopol 974P as the previous study, the in vitro release revealed that the developed formulation released brimonidine for up to 8 h, while the marketed ophthalmic drops were released in a short time [59]. Less frequent administration was also able to prevent systemic side effects, as mentioned previously. In agreement with Pang et al., the in vivo study also showed excellent IOP-lowering activity in the in situ gel with a 45.71 ± 4.72% reduction up to 8 h, while marketed drops reduced 13.38 ± 4.42% up to 2–3 h only. An ex vivo transcorneal permeation study that was performed on goat cornea further clarified that the in situ gel system exhibited excellent drug penetration for up to 5 h with a permeation flux value of $28.25\pm 1.1\;\mu g/{\mathrm{cm}}^{2}h$. As explained, the carbomer is able to gel and interacts with the mucin layer of the corneal via hydrogen interaction, resulting in a thin film that is able to resist the blinking forces and the tears washout of the eyes [79]. Likewise, Karuppaiah et al. also developed brimonidine in situ gel with Carbopol 940 that could release brimonidine 3 h longer than the marketed ophthalmic eye drops [60]. Burst released from the formulation was hardly observed throughout the study, while that being said, it usually happened when the drug was not fully entrapped in the gel matrix. In contrast, the formulated in situ gel was reported to have first-order release rate, while Barse et al. reported a zero-order release rate. This explained that the in situ gel formulation released drug could either on the drug concentration or be independent of the drug concentration, which varies by the amount of gelling polymer used and the compactness of the polymer matrix.

Dorzolamide does not effectively manage open-angle glaucoma but can be considered when intolerant to primary treatment [80]. Kouchak et al. present the efficacy of the dorzolamide IOP-lowering profile in solution, marketed drops (Biosopt^®^), and in situ gel formulation utilizing Carbopol 940NF [61]. The lowering effect was 8.05%, 15.9%, and 26.4%, corresponding to dorzolamide solution, Biosopt^®^, and in situ gel formulation. Overall, it showed 1.7-fold and 3.3-fold effectiveness in lowering IOP compared with Biosopt^®^ and dorzolamide solution, respectively. In agreement with all the studies, the incorporation of HPMC in the formulation further aids the retainability of the formulation on the corneal. Other than the hydrogen interaction of the carbomer, hydrogen bonding between the hydroxyl groups of HPMC with the amide group of mucins was also discovered to further enhance the mucoadhesive properties [81]. Thus, it enhanced the bioavailability of the drug and achieved the therapeutic effect.

##### Ocular Bacterial Infections

Bacterial conjunctivitis is a contagious ocular infection that manifested symptoms such as mucoidal discharge, irritation, burning, and stinging sensation that affect the vision of a patient.

Sheshala and colleagues developed a sulfacetamide sodium in the pH-responsive in situ gel by comparing two carbomer grades, namely Carbopol 934 and Carbopol 940 in combination with HPMC to minimize the acidity of carbomers as well as a viscosity enhancer [62]. The study found that Carbopol 940 had a higher viscosity compared to the Carbopol 934 with the feature of higher cross-linking intensity in Carbopol 940. The formation of viscoelasticity gel was clarified by the hydrophobic aspect of the carbomer backbone. An increment in cross-linking density indicated that high amounts of polymeric chains create hydrophobic interchain aggregation. When the external pH increased, a high ionization of functional groups will be achieved, thereby increasing the repulsion forces among the polymer. The higher cross-linking ultimately strengthens the polymeric network and thus allowed better drug-sustained release. In this case, the adapted formulation revealed a sustained release for up to 8 h at a zero-order release rate in a non-Fickian manner. The author also compared the formulated in situ gel with the marketed formulation (Albucid^®^ 10%) and found out that there was no substantial difference in antimicrobial activity (*p* > 0.05), which suggested that the formulating sulfacetamide into in situ gel would not compromise its antimicrobial activity.

Similarly, Sethuraman and co-authors also established ofloxacin with the same pH-responsive approach in the in situ gel [63]. The prototype formulation demonstrated excellent gelling capacity in contact with stimulated tears fluid (STF) with pH 7.4 at room temperature. The in vitro release study revealed that the prototype formulation has the highest drug released over 8 h, which was in agreement with Sheshala and co-authors. The prototype in situ gel also obeyed the zero-order release rate and followed the non-Fickian diffusion model, suggesting that the drug molecule that was entrapped in the gel matrix was released by diffusion and erosion of the polymeric matrix. Furthermore, Sethuraman et al. additionally carried out an ex vivo release study using the goat cornea membrane on a Franz diffusion cell that showed a maximum drug released of 95.57% at the end of the 7th h. The author performed an antibacterial activity by using *Escherichia coli* and *Bacillus subtilis* strains. The prototype formulation showed good ZOI with no substantial difference than the standard, which indicated that ofloxacin efficacy was not affected during formulating. However, both studies did not further evaluate the mucoadhesive strength and safety of the antibacterial in situ formulations.

Another ofloxacin formulation was designed concomitantly with diclofenac in the in situ gelling systems that covered the permeability and safety testing, which was not done in the previous studies [64]. Similar to Dubey et al., this research used combination therapy to treat bacterial conjunctivitis and prevent the progression of ocular inflammation. Carbopol 934 was utilized in conjunction with HPMC in this research. An in vitro release study demonstrated that the designed formulation could release both ofloxacin and diclofenac up to 8 h in the same manner as the studies discussed above. However, an initial burst released was reported in this study due to the low viscosity of the formulation at the start, but it decreased as the formulation fully transformed into a gel. An ex vivo drug permeation test using goat cornea was performed and revealed that the accumulated drug permeated was 86.96 ± 4.64% and 89.97 ± 4.92% for ofloxacin and diclofenac, respectively, 8 h from the designed formulation. Antimicrobial efficiency showed ≥86% efficiency against *Staphylococcus aureus* compared to standard ofloxacin solution. In addition, ocular irritation study was performed in this literature as per Draize protocol by using albino rabbit models. None of the rabbits showed any clinical signs of irritation or abnormalities on the corneal, iris, or conjunctiva.

Alternatively, the use of PCP in developing a pH-responsive in situ gelling system was proposed by Prasanth et al. in formulating levofloxacin hydrochloride [65]. The Noveon^®^ AA-1 PCP is an acrylic acid polymer conjugate with divinyl glycol that possessed great mucoadhesive features [82]. The blending of PCP with HPMC has established a clear transparent solution with a pH value of 6.98 approaching the physiological pH of the eye. The formulation demonstrated excellent gelling capacity and remained on the ocular surface for an extended period. The released profile was displaying Fickian diffusion for up to 8 h. Prasanth et al. explained that the extended drug released reflected the relaxed diffusion of the polymer network through micropores. The combination with HPMC in the formulation had potentiated gel matrix construction, resulting in a constant release rate [65]. Antimicrobial studies also performed in *Staphylococcus aureus* and *Pseudomonas aeruginosa* showed promising efficacy. Furthermore, the irritation test had demonstrated no ocular damage or abnormal clinical signs on albino rabbits’ eyes.

Apart from synthetic polymers, natural polymer *Terminalia arjuna* (*T. Arjuna*) bark resin also possesses excellent gelling properties in response to pH stimuli. Noreen et al. employed *T. arjuna* gum blended with sodium alginate to synthesize in situ pH-responsive gel carrying moxifloxacin hydrochloride in managing bacterial conjunctivitis [66]. The optimized formulation containing *T. arjuna* gel and sodium alginate has a clear, viscous appearance with suitable ocular delivery consistency. Sodium alginate was added to improvise the viscosity of the formulation by interacting with the divalent-cation presence in the tear fluid. An in vitro release study displayed sustained release over 12 h without significant burst released. Additionally, the optimized formulation was further compared for its drug permeability with a marketed brand (Ocumox^®^) by using goat cornea. The results showed excellent drug permeability in developed in situ gel with a 2.3-fold increment in permeated drugs. As explained, the permeability improved vastly when the formulation was able to retain on the corneal surface with optimal viscosity, thus permitting the drug to penetrate the corneal barrier and reach the targeted site of infection. The biocompatibility of the formulation was further clarified by undergoing several tests. Firstly, the cytotoxic effect on MCF 7 breast cancer cell line had displayed a negligible cytotoxic impact, which indicated that *T. arjuna* may be safe to use on human eyes. Secondly, the biocompatibility was tested on HET-CAM, and rabbit models under modified Draize eye protocol revealed that the in situ gel with *T. arjuna* was non-irritant for ocular application. On top of that, the antimicrobial effectiveness tested on *Escherichia coli*, *Staphylococcus aureus*, *Bacillus subtilis*, and *Salmonella typhi* disclosed comparable efficacy with Ocumox^®^. In brief, natural polymer *T. Arjuna* can also achieve gelling ability as a synthetic polymer and provide an extended released profile that is harmless on the eyes. Moreover, future cytological and other analyses were recommended to explore the uptake mechanism at the molecular level further.

Interestingly, merging both nanocarriers with the in situ gelling systems was also found in a few studies. Allam et al. developed a nanosized niosome loaded in the pH-responsive in situ gelling systems to deliver vancomycin in methicillin-resistant Staphylococcus aureus MRSA patients with ocular infection [67]. Vancomycin is an excellent drug in eradicating MRSA infection. However, due to instability in the aqueous medium, vancomycin ophthalmic ointment and reconstituted vancomycin acid solution have been practically used, resulting in blurred vision and ocular irritation upon instillation. In addition, vancomycin is a time-dependent antibiotic that requires prolonged residence time on the infected ocular site. Therefore, to improve its permeability and prolong its residence time, vancomycin was first entrapped in niosome and mixed with in situ gel to prolong the residence time. Niosome was successfully prepared from the equimolar ratio of Span 60, Tween 40, and cholesterol. The niosome has shown the best entrapment efficacy of vancomycin and was subsequently loaded in the optimized in situ gel containing Carbopol 934P and HPMC. The acquired formulation showed excellent gelling capacity, viscosity, pH, and mucoadhesiveness. A slight increment in the hydrodynamic size of the niosome was observed over a two-month period after being united into in situ gel. However, the entrapment of vancomycin remained stable for the two-month period. The increase in niosomal size after integration into in situ gel may be due to the absorption of some polymers on the surface of the niosome via a weak hydrogen bonding interaction. Fortunately, the overall dispersion was not significantly affected. The release kinetics of the developed niosomal in situ gel displayed excellent sustained release up to 24 h. The cumulated drug released of niosome alone and in situ gel alone had a greater amount than the acquired formulation having 39.2 ± 3.2% only due to the double barrier that existed in the formulation. The release mechanism was shown to obey the Higuchi diffusion model, whereby the drug molecule diffused through the niosome bilayers and the gel matrix before being released into the medium. Antimicrobial testing was further evaluated by comparing to vancomycin solution. An in vitro with agar diffusion method showed no substantial difference, but the minimum inhibition concentration (MIC) was 2-fold effective against the MRSA strain after 24, 48, and 72 h (*p* < 0.05). On the MRSA-infected rabbit model, the niosomal in situ gel demonstrated a 2.5-fold reduction in the colony-forming unit (CFU) compared with vancomycin solution. The author clarified that the improvement may be due to the interaction between the niosome and the microorganism [67]. The entrapment of niosome in the gel matrix provided a longer contact time for the niosome to fuse with the bacterial cell wall. Consequently, the permeability of the vancomycin could be enhanced. Allam et al. also highlighted that the rapid drug release observed during the initial release phase had allowed adequate concentration of vancomycin to inhibit early colonization of the bacteria. Meanwhile, the sustained release phase further maintained the antibacterial activity for a prolonged period [67]. The biocompatibility test assessed with albino rabbits had shown the absence of ocular irritation, redness, swelling, or mucoidal discharge, representing that the niosomal in situ gel was safe to apply on eyes.

**Figure 5:**
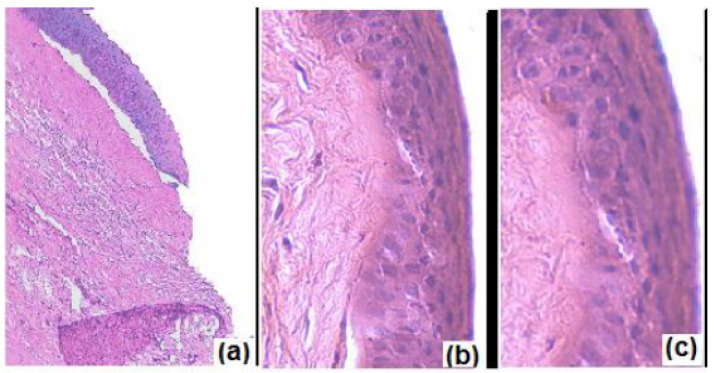
Histological cross-section of excised goat cornea stained with hematoxylin–eosin after treated with (**a**) isopropyl alcohol as the positive control; (**b**) normal saline as the negative control; and (**c**) a developed chitosan nanoparticulate in situ gelling system [68]. A noticeable difference comparing both developed formulations with positive control is the widening of intracellular spaces with the distortion of superficial epithelial cells, which highlights that the developed formulation was generally tolerable on the goat cornea.

Another study that utilized a dual system in formulating norfloxacin was performed by Upadhayay et al. using chitosan nanoparticle loaded in the in situ gel system [68]. Chitosan was selected in formulating ocular drug delivery systems due to its non-toxic, biocompatible, and mucoadhesive properties. It is a cationic polysaccharide that is solubilized in an aqueous solution at pH < 6.5 [83]. It is capable of interacting with tight junctions found in the epithelium layer of the corneal, vagina, esophagus, stomach, small and large intestine, which during protonation made it act as a paracellular permeability enhancer [83]. An opalescent chitosan nanoparticle suspension containing chitosan and sodium tripolyphosphate was developed and showed outstanding characteristics of high drug entrapment efficiency, homogeneous particle size, and positive zeta potential, and it was also slightly acidic, which is a great medium for norfloxacin solubility [84]. Sodium tripolyphosphate was added to form cross-linkage with chitosan for better drug entrapment. The author pointed out that the concentration for both chitosan and sodium tripolyphosphate should be moderate. Too low concentration would fail at cross-linking with chitosan, while too high concentration would create rigid interaction, leading to poor swelling ability and erosion rate, which in turn hindered the drug released from the nanoparticle. The integrated chitosan nanoparticle with the in situ gel system had been shown to slightly alter the spherical boundaries due to the gel matrix stressing on the nanoparticles. The developed nanoparticulate in situ gel formulation that contained Carbopol 934P exhibited sustained release up to 12 h, which obeyed the Higuchi release model and was in agreement with previous study. Although the nanoparticle showed an initial burst release, this issue was resolved after formulation in the in situ gelling system, where polymers strengthen the barriers for the drug to release. The nanoparticulate in situ gel displayed a mucoadhesive strength that was eight times higher than the ocular shear force, as chitosan and carbomer both possessed bioadhesion properties. The developed formulation was tested for antibacterial activity on *Staphylococcus aureus* and showed no significant different in comparison with the marketed formulation. Furthermore, the author carried out a biocompatible test to ensure that the developed formulation was safe to instill on the ocular site. HET-CAM was carried out to test ocular irritation and revealed no signs of membrane discoloration or hemorrhage. However, at the 12th h, one test egg shows visible membrane discoloration. In the cross-section of goat cornea (Figure 5), the formulation did not show any sign of tissue damage as compared with the positive control, which showed distorted superficial epithelial cells. It was concluded that the developed formulation was slightly irritant, which was attributed to the presence of Carbopol 934, which shifted the pH drastically from acidic to neutral.

##### Ocular Fungal Infections

Fungal keratitis is a corneal infection commonly caused by *Fusarium, Aspergillus,* or *Candida* species [85]. A prolonged duration of treatment is vital to avoid the recurrence of infection. Antifungal itraconazole formulated by Jaiswal et al. incorporated polymeric micelle into an in situ gel system and showed impressive results [69]. Both polymers have a similar low carboxy methyl cellulose (CMC) value, which displayed better stability in the diluted medium. Pluronic F-127 was selected to optimize the polymeric micelle with a lower polydispersity index, micellar size of 79.99 nm, and highest entrapment efficacy of 91.32 ± 1.73%. Having the lowest polydispersity index indicated that the formulation acquired homogeneous micellar size distribution. Next, the selected polymeric micellar formulation was integrated into the optimized in situ gel with Carbopol 943P. The ex vivo transcorneal permeability of the micellar in situ gel was tested and exhibited 5.6-fold higher cumulated drug permeability than itraconazole suspension, while in terms of permeability flux, that of the prototype formulation was 3-fold higher compared to the marketed drop (Itral^®^). It is noteworthy that the polymeric micelle allowed inter-diffusion of the medication across the corneal epithelial layer through transcellular diffusion, thereby remarkably improvised drug permeation. The formulation fit into various kinetic models obeying a zero-order release rate for up to 8 h. Such sustained release implied that the drug released from the shell of the polymeric micelle was rapidly replenished by the reservoir drug available in the micellar core independent of the drug concentration. The antifungal efficacy of the micellar in situ gel showed a better ZOI compared with Itral^®^ in *Candida* inoculated agar. The author clarified that the significant improvement was mainly due to the interaction between the negatively charged mucin layer with carbomer by hydrogen bonding, thereby permitting the formation of a thin film that prolonged the residence time of the micelle to release the medication in the cul de sac. *Fusarium* and *Aspergillus* were recommended for further validation of the antifungal spectrum of this promising formulation. HET-CAM testing was performed, wherein formulated micellar in situ gel showed faintly visible membrane discoloration in one tested egg at 480 min and another egg at 720 min. Thus, the mean score at the end of 720 min of the HET-CAM test was 0.67, which suggested it was a mild irritant. Likewise, the irritancy may be due to the shift of pH value in the formulation containing Carbopol from 3.20 to 6.84.

##### Ocular Viral Infections

As discussed previously, HSK is one of the leading causes of blindness. The current treatment available includes acyclovir ointment, ganciclovir gel, and trifluridine eye drops. Kumar and colleagues carried out the most recent literature in formulating valacyclovir in the pH-responsive in situ gel system by utilizing Carbopol 940 and showed sustained release of the formulation [70]. The in vitro diffusion of valacyclovir performed in Franz diffusion cell revealed that the drug followed zero-order release for up to 8 h.

Previously, Kapadia et al. also developed acyclovir with niosome entrapped in a pH-responsive hydrogel. Instead of Carbopol 940, Carbopol 934 was incorporated as the gelling agent [71]. Similar to Allam et al., the optimized niosome formulation was determined and added into optimized in situ gel. The author compared Span 20 and Span 60 combined with cholesterol and discovered that the ratio 7:6 of Span 60: cholesterol possessed the acceptable entrapment efficacy. The author explained that the entrapment efficacy varied with the ratio of surfactant to cholesterol. As the cholesterol reached the maximal concentration, it raised the microviscosity of the membrane, thereby forming a more rigid bilayer with the surfactant. Consequently, it disrupted the regular bilayers and lost its drug entrapment ability. Span 60 was selected over Span 20 due to its longer alkyl chain length, which ultimately contributed to better drug entrapment. Interestingly, the author compared the release kinetics of (D1) acyclovir in niosome only entrapped in hydrogel, and (D2) acyclovir in both niosome and hydrogel systems revealed that the released was much slower in D1 compared to D2. Kapadia et al. also carried out biocompatibility testing under Draize protocol and reported that the combination system was safe with no irritancy recorded in both D1 and D2.

##### Other Ocular Diseases

Other ocular diseases that exploited pH-responsive strategy in the in situ gelling systems included allergic conjunctivitis, anti-inflammatories, and a few unorthodox medicines. The particulars of the literature will be covered briefly subsequently.

Allergic conjunctivitis, also referred to as ocular allergy, is an ocular inflammation in response to allergens such as pollen or mold spores. Recent researchers have tried to develop and evaluate the efficacy of several antihistamines in the in situ gelling systems. Naphazoline and Antazoline in situ gels developed by Shetty et al. composed of Carbopol 940 as the pH-responsive gelling agent together with HPMC were shown to be superior [72]. The in vitro release of marketed eye drops in was reported up 3 h, while the in situ gel formulation could be released up to 8 h. The release mechanism of the prototype formulation for both naphazoline and antazoline obeyed the Korsemeyer–Peppas model and fit in the Fickian diffusion model. The in vivo drug bioavailability of the formulated in situ gel compared with marketed naphazoline and marketed antazoline showed that the AUC in formulated in situ gels was higher than that of the marketed formulation [72].

In another similar study, Carbopol 974 was utilized as a gelling agent in formulating olopatadine hydrochloride to treat allergic conjunctivitis. Two optimized formulations were selected in this study with (F3) 0.1% *w*/*v* Carbopol 974 and 1.0% *w*/*v* HPMC E50LV and (F7) 0.3% *w*/*v* Carbopol 974 and 0.6% *w*/*v* HPMC E50LV with appropriate gelling capacity within a pH increasing environment after 3–4 h [73]. Both optimized formulations have a free flow characteristic in slightly acidic pH. Simultaneously, the viscosity of F7 was 2.3 times higher than F3 when undergoing physiological pH conditions, which was attributed to the higher carbomer content in the F7 formulation. Viscosity affected the instillation of drops and immediately transformed into a thin precorneal film with good spreadability in the ocular surface and ultimately improved the duration of contact time on the eyes. The release kinetics was studied using a cellophane membrane and goat cornea, and the researchers discovered that the release rate was slower in goat cornea than the cellophane membrane. In addition, the author also compared F3 and F7 with marketed olopatadine hydrochloride eye drops. The marketed eye drops released drugs up to 6 h while the formulated in situ gel released drugs up to 12 h. An ocular irritation study revealed that both F3 and F7 did not show any sign of irritation or abnormal ocular sign on rabbit eyes, which indicated the formulation was safe.

Non-steroidal anti-inflammatory naproxen is formulated in the in situ ophthalmic gel system for the maintenance of mydriasis during cataract surgery as well as controlling post-surgery inflammatory response. Dawood et al. compared the grade of HPMC K40 and HPMC K100 to achieve optimal gelling capacity in combination with Carbopol 940 [74]. The optimized formulation appears to be translucent dispersion due to the precipitation of HPMC at elevated temperatures. HPMC K100 has a higher density of hydrophobic methyl group compared to HPMC K40, hence having a more polymeric network and thereby better gelling capacity. The in vitro release study of HPMC K100 did not show significant burst released as compared to HPMC K40 due to the better consistency of the formulation when mixed with Carbopol 940. The optimized formulation containing Carbopol 940 and HPMC K100 demonstrated first-order release kinetics up to 3 h, which fitted into a Fickian diffusion model. The amount of drug released from a swelling matrix has relied on the drug dissolution and diffusion and solid drug translocation in the gel during swelling of the polymers [86]. The expansion of the polymer matrix permitted the movement of the dissolved drug out of the formulation until the glassy core of the hydrated layer had diminished. Lastly, the author concluded that the formulation was safe, as an in vivo eye irritation test displayed the absence of abnormal clinical signs in corneal, iris, and conjunctiva in rabbit models.

**Figure 6:**
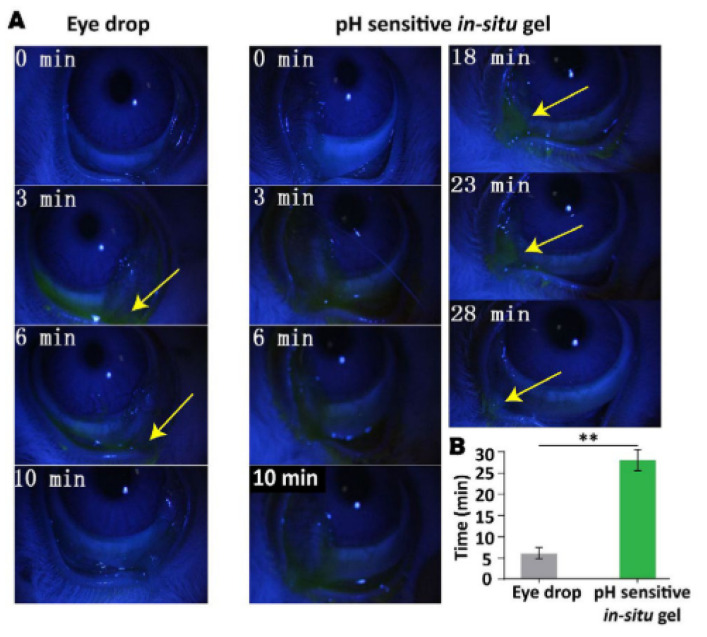
Ni et al. revealed (**A**) in vivo dwelling time of marketed bear bile eye drops and advanced formulation displayed by fluorescence-gel formulation, the residence time was remarkably longer in advanced formulation compared to marketed drops; (**B**) the retention time of marketed bear bile and advanced formulation, where ** *p* < 0.01 [75].

**Figure 7:**
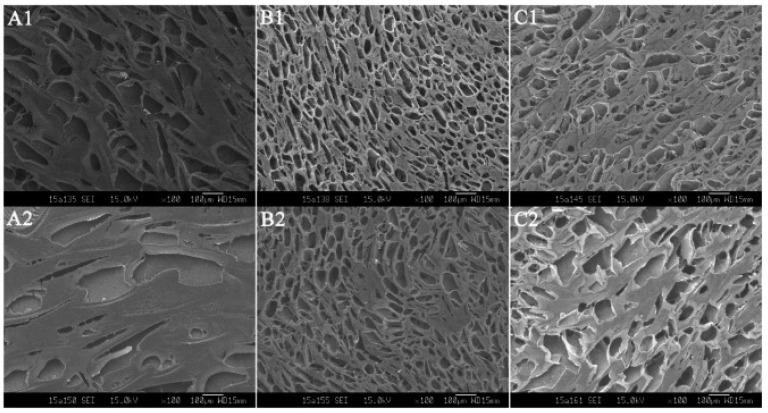
Ni et al. displayed the morphology of developed bear bile-loaded pH-responsive in situ ocular gels. (**A1**): G1, pH 5.0; (**A2**): G1, pH 7.2; (**B1**): G2, pH 5.0; (**B2**): G2, pH 7.2; (**C1**): G3, pH 5.0; (**C2**): G3, pH 7.2 [75].

Traditional Chinese Medicine (TCM) is an alternative, complementary treatment in the modern world involving acupuncture and herbal medicine to adjust the imbalance of yin and yang. Recent studies incorporated bile and baicalin in the in situ gelling systems to improve its medicinal advantage as a complementary medicine treating ocular diseases. The significant component of bear bile, taurousodeoxycholic acid (TUDCA), has shown stunning therapeutical results in the retina [75]. Thus, it is beneficial to improve the delivery of TUDCA in combating ocular disease, as mentioned above. Ni et al. acquired TUDCA in the in situ gel employed with Carbopol 974 [75]. The addition of bear bile attenuated the gelling capacity of Carbopol/HPMC due to the presence of ${\mathrm{Ca}}^{2+}$, ${\mathrm{Mg}}^{2+}$, and ${\mathrm{Zn}}^{2+}$ found in bear bile. The author overcame this by adjusting the amount of excipient in the in situ gel formulation. Moreover, TUDCA happened to have a low log *p* value, which implied that the hydrophilicity of the bear bile could limit the permeation of the drug through the corneal. Hence, a prolonged corneal retention time is required for improving the permeation of TUDCA. The in vivo dwelling time was done by administering fluorescence-gel formulation, which showed that the prototype formulation demonstrated a longer residence time in the conjunctival sac up to 28 min, while the marketed eye drops fluorescence disappeared after 9 min, as showed in Figure 6. In addition, the prototype formulation was found to have slower gel erosion and sustained release up to 160 min. The sustained release feature was not only due to the optimal amount of carbomer and HPMC but also contributed by the 3D porous network structure with denser pores, which allowed slow gel erosion and stable drug released from the matrix, as displayed in Figure 7. The biocompatibility examination showed that no irritation or abnormal ocular signs were observed, confirming that the developed in situ gel was non-irritant.

Apart from this, baicalin has been used for decades in TCM and is officially listed in the Chinese Pharmacopoeia with its wonderful medicinal benefits. Baicalin is a flavonoid purified from the medicinal plant *Scutellaria baicalensis* Georgi having anti-inflammatory, anti-Chlamydia, antibacterial, anti-oxidative, and anti-cataract effects on the eye. Wu and colleagues formulated baicalin in situ ophthalmic gelling systems by employing the same polymer as the previous study [76]. The designated formulation displayed sustained released up to 8 h with a first-order release rate. The release mechanism obeyed non-Fickian diffusion, which implied that the formulation released baicalin by diffusion and erosion. Pharmacokinetic studies in aqueous humor also found out that the AUC of the designated formulation was 6.1-fold higher than the control solution (*p* < 0.01). However, unlike Ni et al., the formulated in situ gel was not compared with the marketed formulation. In agreement with Ni et al., the biocompatibility examination demonstrated no irritation nor abnormal ocular signs in rabbit eyes, confirming that the gel was non-irritant.

### 3.3. Ion-Responsive In Situ Ocular Gelling Systems

Ion-responsive in situ ocular gelling systems, otherwise known as ion-activated or ion-sensitive systems, exhibit their sol–gel transition due to cross-linking with monovalent (Na^+^) or polyvalent cations (Ca^2+^ and Mg^2+^) in the lachrymal fluids [2,4]. Recent approaches to deliver therapeutics using the ion-responsive gelling system for improved efficacy have been summarized in Table 3.

#### 3.3.1. Recent Research on the Application of Ion-Responsive In Situ Ocular Gelling Systems

##### Ocular Hypertension (Glaucoma)

Brinzolamide is one of the treatments for glaucoma. However, there are several limitations associated with brinzolamide suspension available commercially, such as eye burning sensation, stinging, pain, taste aversion, and cost. To overcome these limitations, Sun and Zhou developed an ocular brinzolamide in situ gel using gellan gum [87]. The optimized in situ formulation (Gel B) showed in vitro sustained drug release where 92% of the drug was released over 12 h compared to liquid (2 h). This was because the ion exchange between gellan gum and simulated tears fluid had led to in situ gelations that slowed down the diffusion and erosion of polymers and drugs, thereby sustaining drug release [87]. The in vivo pharmacodynamics studies compared the IOP in rabbits treated with either normal salines, brinzolamide dispersion, or brinzolamide in situ gel. According to the results, brinzolamide dispersion reduced IOP by 27% after 1 h administration, but the IOP quickly rebounced over baseline value after 6 h [87]. In contrast, the IOP of brinzolamide in situ gel lowered IOP by 18.2% after 1 h and remained below baseline value after 6 h. This was because the in situ gel enhanced the drug ocular retention and local drug availability [87]. In vivo irritation studies showed slight conjunctival hyperemia in the rabbits treated with in situ gel, but it was not significantly different from the control (marketed suspension, Azopt^®^) [87].

Based on a similar concept, Bhalerao et al. prepared brinzolamide in situ ocular gel with gellan gum [88]. Bhalerao et al. had successfully dissolved brinzolamide using a solubilizing agent (dimethyl sulfoxide, polyoxyl 35 castor oil) before mixing it with gellan gum. Soluble brinzolamide showed more proficient drug transport through the corneal barrier than the marketed suspension [88]. The prototype in situ gelling formulation showed instant gelation (within 10 s upon contact with lacrimal fluid) due to ions-induced polymer aggregation [88]. The formation of gel with optimal viscosity had hindered rapid nasolacrimal clearance, thereby providing extended ocular residence time (16–24 h) compared with suspension (4.9 h). Subsequently, time-dependent polymer erosion and dissolution were responsible for further prolongation of drug release (43 h) [88]. In addition, a significant in vivo IOP reduction was observed due to higher drug exposure, as evidenced by an AUC _(_∆_IOP vs. t)_ that was 2–9 folds greater than the marketed product. Thus, the same therapeutic outcome could be achieved at a reduced dosing frequency (once daily for in situ formulations vs. thrice daily for marketed suspension). Moreover, prototype in situ formulations were well-tolerated after evaluating ocular toxicity using NZW rabbits. In short, this novel brinzolamide in situ gelling system appears to be stable and efficacious for sustained ocular drug delivery to treat glaucoma. It is a cost-effective option because it requires fewer doses per day. Moreover, Bhalerao et al.’s formulation appear better than Sun and Zhou’s formulation by avoiding the issue of non-uniform drug dispersion [87].

Apart from brinzolamide, timolol maleate is another antiglaucoma agent that blocks β-receptors in the ciliary body [89]. Yu et al. prepared an ion-sensitive in situ ophthalmic gel consisting of timolol maleate liposomes to enhance its ocular bioavailability and histocompatibility [89]. Compared to the conventional timolol eye drops, liposomal timolol yielded a 1.93-fold higher in vitro apparent permeability coefficient (P_app_). This could be contributed by the liposomal lipid vesicles, which were biocompatible with corneal epithelial cells, leading to increased solubility and easy transport of the encapsulated drugs through the corneal [89]. Timolol maleate-loaded liposomal in situ gels (TM L-ISG) showed sustained in vitro drug release. Furthermore, TM L-ISG demonstrated extended retention time on the corneal surface compared with the eye drops. TM L-ISG achieved its peak effect in 1 h and the effect lasted for 5 h, owing to greater cornea transmittance and longer permanence time [89]. More importantly, TM L-SG showed no significant difference with 0.9% sodium chloride in eye irritation studies, and the histological observation found no inflammatory cell infiltration, hemorrhage, or necrosis. This could be due to the biocompatible nature of liposomes and gellan gum [89].

##### Ocular Bacterial Infections

Generally, the treatment of ocular bacterial keratitis and conjunctivitis requires topical antibacterial solution. Gatifloxacin is an antibacterial agent against staphylococcus and streptococcus, which are the most common pathogens that cause bacterial eye infections [90]. However, gatifloxacin absorption is limited by the short residence time of its eye drops. As such, Kesavan et al. examined the effectiveness of gatifloxacin ion-activated mucoadhesive hydrogel to treat bacterial keratitis [90]. The researchers employed gellan gum or sodium alginate as the gelling agent and sodium carboxymethylcellulose as the mucoadhesive enhancer. In this study, two optimized formulations (GG5 and GS5) had successfully shown retardation in drug release rate and excellent mucoadhesive property. The stroma of NZW rabbits was inoculated with *Staphylococcus aureus* colony for the purpose of antimicrobial efficacy test. The results revealed an earlier symptoms improvement in the group treated with ion-activated gel systems as compared to marketed eye solution (*p* < 0.05). These results could be explained by the gel’s mucoadhesive strength, resulting in enhanced precorneal contact time of the gelling systems, in turn impeding drug elimination via nasolacrimal drainage [90]. The ocular irritation studies showed an absence of inflammation, conjunctival congestion, swelling, or discharge in the rabbits’ eyes [90].

Likewise, levofloxacin eye drops used for bacterial conjunctivitis result in rapid elimination from precorneal space. To overcome this shortage, Chandran and colleagues designed an in situ gelling levofloxacin hemihydrate using ionic-dependent gellan gum [91]. In the observation, the in situ formulations displayed in vitro prolonged drug release (18–24 h), which was ascribed to the three-dimensional network gel structure formed after gellan polymers interacted with cations in the STF, thereby slowing and sustaining drug release from the gelling base [91]. According to the in vivo study, the in situ gelling levofloxacin showed 2.7-fold higher ocular bioavailability. In situ gelling levofloxacin maintained MIC for 8 h compared to marketed product (4 h). The author attributed the in vivo performance to the in situ gelation in the cul de sac, which had impeded the precorneal elimination of levofloxacin by rapid tear drainage and blinking of eyelids [91]. Lastly, the in situ gelling system was well-tolerated and did not indicate irritation or toxicity based on Organisation for Economic Co-operation and Development (OECD). Again, in situ gelling ocular delivery shows better performance to treat bacterial eye infections.

##### Ocular Fungal Infections

Natamycin is the only Food and Drug Administration (FDA)-approved ophthalmic antifungal agent for treating fungal keratitis. Due to its low solubility, corneal permeability, and residence time, current therapy requires multiple dosing (a drop every 1–2 h) for more than a month. To improve ocular antifungal therapy, Janga et al. formulated natamycin bilosomes ion-sensitive in situ hydrogel [92]. The optimized formulations, natamycin-bilosome-2 (NB-2) and in situ gel (NBG-2) made from gellan gum, exhibited significantly higher in vitro transcorneal flux and P_app_ compared to a control (*p* < 0.05). This could be attributed to bilosomes’ enhanced drug penetration through corneal lipid fluidisation [92]. In vivo ocular disposition studies showed that in situ gel delivered a higher drug concentration to all ocular tissues at a fixed time (6 h later) due to prolonged drug delivery [92]. The outcomes were consistent with Yu et al. [89] who reported that in situ hydrogels facilitated higher adhesion to biological membranes via cross-linking with glycoproteins moieties of the epithelial cells, hence longer residence and drug delivery [92]. Additionally, the topically applied colloidal bilosomes showed potential penetration into posterior eye tissues, such as retina choroid [92]. The in vitro cytotoxicity test performed on human corneal limbal epithelial cells suggested that the optimized formulations were well tolerated, as the cell viability results (100.5 ± 4.5% and 110.7 ± 5.6%) were not statistically different from saline solution [92]. These results confirmed the advantages of ion-sensitive in situ hydrogels of natamycin bilosomes over conventional ophthalmic solution.

##### Ocular Viral Infections

Sustained ocular drug delivery of antiviral agent for treating HSK is the main focus of this review article. Interestingly, kappa-carrageenan, an ion-sensitive polymer, exhibited antiviral effect on herpes virus [95]. With this advantage in mind, Li et al. evaluated ophthalmic acyclovir incorporated into kappa-carrageenan for ocular herpetic infection [93]. HPMC was used as viscosity enhancer while hydroxypropyl-β-cyclodextrin (HPBCD) functioned as a penetration modifier [93]. The in vitro release demonstrated that the commercial eye drops released almost 95% acyclovir immediately after 0.5 h [93]. In comparison, acyclovir in situ gel released 17% drug after 0.5 h and then 80% drug after 6 h [93]. The results supported that an in situ matrix exhibited a significant sustained-release effect without burst release [93]. Owing to swelling mechanism of gel matrix, the drug diffused into the affected tissue at a controlled rate [93]. The presence of 2% HPBCD caused 2.16-fold higher acyclovir P_app_ compared with conventional eye drops (*p* < 0.05) [93]. Compared to other permeation enhancers, HPBCD did not adversely affect the corneal hydration level and blinking count [93]. Ocular irritation assay reported no abnormality of the conjunctiva, corneal, or iris [93]. Summing up, Li et al. demonstrated that acyclovir ion-activated in situ ophthalmic gelling systems possess pronounced ex vivo drug permeation and in vitro sustained drug release [93]. Nevertheless, further study on the in vivo antiviral efficacy and inhibitory concentration (ID_50_) of this formulation may be required in the future to examine the dosing regimen.

##### Other Ocular Diseases

Postoperative pain and inflammation in the cornea are common side effects following eye surgeries, which can be alleviated using nepafenac, a non-steroidal anti-inflammatory drug (NSAID). Due to its water-insoluble nature, nepafenac is only marketed as ocular suspension, Nevanac^®^. However, suspensions tend to cause foreign body sensation and lacrimation, limiting drug availability at the ocular site. In an attempt to improve the sustained release and ocular residence time of nepafenac, Shelley et al. formulated a nepafenac ion-activated in situ gel, using sodium alginate (Protanal PH 1033) as the gelling agent, and HPBCD as the drug solubilizer and permeation enhancer [94]. The author found that the Protonal PH 1033 was the ideal gelling agent because it formed strong gel at low concentration due to its high guluronic acid content [94]. All formulations displayed sustained release over 24 h without burse release effect. This was because the swelling of sodium alginate controlled drug diffusion from the alginate matrix [94]. The in situ gel systems displayed a significantly higher in vitro diffusion rate compared to Nevanac^®^ (*p* < 0.05). Similarly, the in situ gel systems displayed a ten times higher permeation rate through the cornea compared to Nevanac^®^ (*p* < 0.0001). Accordingly, a significantly higher amount of drug was retained in the cornea. This could be a result of increased solubility of the nepafenac with the help of HPBCD [94]. Ex vivo ocular drug distribution showed prolonged residence time of in situ gel on the porcine cornea and sclera. These observations indicated that the in situ gel formulation would be eliminated from the ocular epithelium more slowly compared to the currently available suspension, which was contributed by the bioadhesive properties and high viscosity of sodium alginate [94]. Interestingly, there was an increased accumulation of nepafenac in the posterior segment of the eye (sclera choroid and retina). It was owing to better drug solubilization and HPBCD’s permeation-enhancing effects [94]. However, this study did not include an ocular irritation study or cytotoxicity test.

### 3.4. Recent Research on the Application of Multi-Stimuli-Responsive In Situ Ocular Gelling Systems

As discussed above, in situ gel formulation is a very effective candidate for active drug delivery to the administration sites. With that being said, the physiological condition of the body, including the ocular site, is very complicated. Therefore, the single-stimuli-responsive in situ gel formulations may not achieve the desired outcomes in drug delivery in certain circumstances. Newer innovations such as the multi-stimuli-responsive in situ gel formulations had been developed to overcome the drawbacks of single-stimuli-responsive in situ gel formulations by making more detailed optimization through more parameters. This section mainly highlights the development of multi-stimuli-responsive in situ gel formulations for the management of glaucoma, bacterial ocular infection, fungal ocular infection, viral ocular infection, allergic conjunctivitis, and eye irritation caused by capsaicin. Multi-stimuli-based in situ ocular gelling systems for the improved ocular delivery of therapeutics have been summarised in Table 4.

#### 3.4.1. Ocular Hypertension (Glaucoma)

Gupta et al. had investigated an ocular in situ gel formulation with a combination of pH-responsive and ion-responsive polymer to improve timolol maleate treatment outcomes for patients suffering from glaucoma. Chitosan functioned as a pH-sensitive gelling agent, and gellan gum functioned as an ion-sensitive gelling agent. The chitosan and gellan gum-based in situ formulation was known as the innovative formulation. The findings revealed that more than 6 folds of timolol maleate was found in the corneal membrane from the innovative formulation in comparison to unbound timolol ocular solution and gellan gum-only based in situ ocular gel. This is because of the outstanding permeability-enhancing characteristics of chitosan. A sterility test had also been done on the innovative timolol maleate in situ ocular gel, and a negative microbial contamination or growth was identified with 2 weeks of incubation. Eye irritation assay was carried out using the HET-CAM test by comparing innovative timolol maleate in situ ocular gel formulation with normal saline. The normal saline achieved a 0 mean score. While the innovative in situ ocular gel formulation scored a 0 up to 1 h after instillation, the mean score slightly increases up to 0.67 after 24 h. The findings obtained revealed that the innovative timolol maleate in situ ocular gel formulation triggered none to mild irritation to the eyes and was well tolerated as ophthalmic drug delivery. Innovative in situ ocular gel formulation illustrated an even distribution over the precorneal surface immediately after instillation in comparison to plain ocular drug solution in gamma scintigraphy images. Other than that, timolol maleate from plain ocular drug solution was cleared very quickly by nasolacrimal drainage and entered into the systemic circulation after 2 h of instillation. On the contrary, the innovative in situ ocular gel formulation was retained on the cornea surface for a longer period and did not enter into the systemic circulation. The main reason is that chitosan has both viscous and bioadhesive properties [77,105]. Furthermore, the final viscosity of the innovative formulation was enhanced, and the more rigid gel was formed due to the dual gelling mechanism [96].

#### 3.4.2. Ocular Bacterial Infections

A combination of in situ ocular gels with a different mode of action was also proven to improve outcomes for the management of bacterial eye infections. Many of the bacteria that invade the human body are also capable of attacking the surface or interior of the eye. These infections may lead to worrying consequences that eventually lead to blindness if left untreated; therefore, physicians often prescribe topical antibiotics for the treatment.

The topical administration of antibiotics through conventional eye drops has a very low ocular bioavailability because of the strong ocular biological barriers. Gupta et al. developed an in situ ophthalmic formulation with the combination of chitosan and gellan gum, which is known as a pH and an ion-sensitive polymer respectively to sustain the release and prolong the retention time of the sparfloxacin on the cornea surface. The chitosan and gellan gum-based in situ ocular formulation was the innovative formulation. Sparfloxacin from an in situ ophthalmic formulation had 41.17% of the total drug released in 2 h after instillation onto the eye, 79.6% drug released after 6 h, and 91.7% drug released after 12 h. On the contrary, the plain eye drops released 75.33% in 2 h after instillation and 89.93% in 4 h after instillation. The finding showed that the in situ ophthalmic gel release was slower than the plain eye drops and had eventually sustained and prolonged the action of sparfloxacin. In addition, ocular irritation of the sparfloxacin in situ ophthalmic formulation was assessed by the HET-CAM test. A mean score of 0 was achieved for normal saline. Sparfloxacin’s innovative in situ gel formulation had a mean score of 0 up to 2 h after instillation, 0.33 in 4 h after instillation, and reached 0.66 up to 24 h after instillation. The outcome revealed that the innovative formulation resulted in none to mild irritation and is generally safe for ocular route drug delivery. Gamma scintigraphic studies were performed on Albino New Zealand rabbits using Tc-99m labeled sparfloxacin in the formulation to obtain in vivo data. The gamma camera images indicated that sparfloxacin was distributed evenly over the whole anterior surface of the cornea for innovative in situ ocular gel immediately after administration, as compared to plain drug solution. Furthermore, the sparfloxacin in the innovative in situ gel was eliminated slowly and was not distributed in systemic circulation, as seen in static images after 5 h. There was a quick fall in the percentage of radioactivity that remained on the cornea for plain drug solution. This indicated that the innovative in situ gel formulation has a lower clearance and stayed on the corneal surface for a longer period. This can be explained by the chitosan becoming more viscous and undergoing gelation as the pH of the external environment increases from 6 to 7 upon administration into the eye due to the buffering action of the lacrimal secretion. The viscosity of gellan gum also increases, which is triggered by ions present in the tear buffer of the eye. This combination leads the formulation to stay for a longer period on the ocular surface that is easily detected by the gamma scintigraphy technique [97].

In addition to Gupta et al., Ameeduzzafar et al. had conducted a research study of thermal, pH, and ion-triggered in situ ocular gelling systems by using chitosan as a thermal and pH-responsive polymer with gellan gum as an ion-responsive polymer for the treatment of bacterial conjunctivitis. The chitosan and gellan gum-based in situ formulation was known as the innovative formulation. Bacterial conjunctivitis is the infection of the conjunctiva caused by several types of bacteria such as *Staphylococcus aureus*, *Streptococcus pneumoniae*, *Haemophilus influenzae*, and *Moraxella catarrhalis* [106]. Some of the less common are *Chlamydia trachomatis* and *Neisseria gonorrhoeae* bacteria. Some of the manifestations include hyperemia, swelling of the conjunctiva, which is associated with discharge (pus) and can lead to eyelids sticking together [107].

The findings of Ameeduzzafar et al. revealed that a very low amount of Besifloxacin (BSF) from ocular suspension formulation was detectable in the aqueous humor of the rabbits in 12 h after instillation, which was the result of rapid lacrimal drainage of suspension formulation. Inversely, the BSF concentration of 105.28 ± 3.35 ng/mL was measured in aqueous humor after 12 h from instillation using an innovative ocular in situ gel formulation. These results indicated that in situ formulation with thermal, pH, and ion mechanisms have sustained release ability. Moreover, the AUC of BSF in an innovative in situ ophthalmic gelling system and the BSF ophthalmic suspension obtained were 3204.36 ± 14.53 ng/mLh and 839.25 ± 23.43 ng/mLh, respectively. The *C*_max_ of BSF from the innovative in situ system was 471.54 ± 10.23 ng/mL, which was approximately 1.63 times higher than the BSF suspension of 289.29 ± 14.72 ng/mL. This is because of the combination of both mucoadhesive properties of chitosan and the outstanding gel strength property of gellan gum, which prolongs the drug retention on the cornea. This ensures there was sufficient time provided to achieve optimum therapeutic agent concentration on the conjunctiva for the management of bacterial conjunctivitis. Next, the BSF goat corneal permeation rate of the innovative in situ gel obtained was about 2.5 times greater than that of the BSF suspension. The better permeation rate of the innovative in situ gel system was mainly attributed to the bioadhesive and penetration enhancing property of both chitosan and gellan gum. An ocular irritation study of innovative besifloxacin in situ gel in comparison with sodium hydroxide (NaOH) and normal saline was performed using the HET-CAM test. The images showed that there was no visible corneal damage observed with the innovative in situ gel formulation and normal saline. However, the sign of corneal damage appeared after the application of NaOH solution. Likewise, in the histopathology test, no signs of damage were discovered in the corneal membrane cells; they were located in the same place with no rupture observed in the corneal section using the innovative in situ ocular formulation compared to the besifloxacin ocular suspension. Therefore, the innovative BSF in situ ophthalmic gel system was found to be free of eye irritation. As for the antimicrobial study, an innovative formulation obtained an inhibitory diameter of 5.2 cm against *P. aeruginosa* and 4.8 cm against *S. aureus* in the incubation time of 24 h, whereas the inhibitory diameter of drug suspension declined to 1.5 cm against *P. aeruginosa* and 1.8 cm against *S. aureus* in the 24 h of incubation time. Hence, the innovative in situ ocular formulation showed a superior inhibitory effect on *Pseudomonas aeruginosa* and *S. aureus* compared to suspension formulation by conducting the agar diffusion test [98].

Other than bacterial conjunctivitis, a combination of in situ ophthalmic gels study had also been carried out by Gupta et al. to treat bacterial keratitis by using levofloxacin as the active ingredient. In this study, chitosan functioned as a pH-stimulated polymer and sodium alginate functioned as an ion-stimulated polymer. The chitosan and sodium alginate-based in situ ocular gel formulation was known as the innovative formulation. The innovative in situ ocular gel formulation exhibited a more gradual release rate of levofloxacin in comparison to plain eye drop formulation. Initially, a burst effect was observed, then gradually approaching slow and followed by constant levofloxacin release. The burst effect might result from the initial migration of the drug particles toward the surface of the matrix. The innovative formulation showed a 34.18% cumulative levofloxacin released in 2 h after instillation. Subsequently, 76.68% was released 6 h after instillation, and 84.08% was released 8 h after instillation. On the other hand, 74.13% cumulative levofloxacin was released in the first 2 h, followed by 97.2% drug release in 6 h from the plain eye drops formulation. Thus, both sustained and control release characteristics were observed in innovative ophthalmic in situ gel formulation [99]. The results could be explained by the interaction of the amino-functional group in chitosan polymer and the aldehyde functional group of alginate polymer. Any optimization of the chitosan and alginate ratio changes the gelation time and subsequently affects the rate of drug release because gel plays an important role in sustaining drug release [108]. An in vitro transcorneal permeation outcome using innovative in situ formulation revealed the well-known bioadhesive and permeation enhancer property of chitosan. A handheld infra-red (IR) camera was used to detect any irritation after instilling levofloxacin plain eye drops and ophthalmic in situ gel formulation by the evaluation of ocular temperature increase. There was no significant rise in temperature recorded after the instillation of both formulations. This revealed that both formulations were non-irritant to the eyes. The acquired gamma camera images displayed that the levofloxacin from the innovative ophthalmic in situ gelling system was distributed uniformly throughout the precorneal area upon application in comparison to plain eye drop formulation. Plain eye drops formulation was drained in a very short amount of time from the corneal region and entered into systemic circulation through the nasolacrimal drainage system. In opposition, innovative in situ gel was cleared at a slow rate and stayed on the corneal surface for a longer time. The nasolacrimal drainage of levofloxacin in innovative in situ gel formulation was less serious because the use of polymer with different mechanisms increases the rigidness of the gel formed on the ocular surface [99].

In addition to conjunctivitis and keratitis, bacterial infection can also occur in the intraocular fluids, including the vitreous and aqueous areas of the eye known as bacterial endophthalmitis. It mainly happens in the postoperative situation or after a serious ocular trauma. The consequences of bacterial endophthalmitis are complete vision loss and persistent ocular pain if treatment is not given immediately. The main treatment plan is an intravitreal injection of antibiotics and vitrectomy in more severe cases. Although both treatment outcomes are promising, both are invasive and traumatizing to some patients. Therefore, Ranch et al. had taken steps to prevent these disadvantages by developing an in situ ocular gel formulation with a combination of Carbopol 940 and gellan gum as pH and ion-responsive polymers. Chloramphenicol, a broad-spectrum antibacterial agent, was chosen because it is able to cover most of the suspected bacteria, and dexamethasone was an anti-inflammatory agent used in this formulation. Based on the result obtained at the in vitro release study, the polymer concentration is one of the parameters that affect the drug release rate. In general, slower chloramphenicol and dexamethasone release rates were recorded as a higher concentration of Carbopol 940 and gellan gum was included in the preparation of the formulation. This is because higher concentration polymer results in a more entangled nature of the polymeric network; therefore, it makes drug particles diffusion across the gel more difficult, slowing the drug release. Furthermore, the entry of water molecules into a higher concentration of polymers formulation was declined, hence lowering the dissolution or erosion rate. Moreover, the density of the gel’s chain structure rises at higher polymers concentrations, which adds more restriction to the active substance movement area. Last but not least, the extent of swelling elevates as the concentration of the polymers increased, thus slowing the diffusion of active drugs from the swollen matrix. In conclusion, the drug release rate could be delayed using both Carbopol 940 and gellan gum as in situ gelling agents [100].

#### 3.4.3. Ocular Fungal Infections

Other than that, ocular keratitis can also be caused by fungal infection from Fusarium species, Aspergillus species, as well as Candida species. After the diagnosis, fungal keratitis is treated with prescription antifungal medicine for several months immediately to prevent further spread of the virus [109]. Therefore, Pawar et al. had established a thermo-ion stimulated in situ ocular gel that enhances the treatment efficacy of voriconazole in fungal keratitis patients. A combination of Pluronic F-68 (PF-68) incorporated into Pluronic F-127 and sodium alginate with different concentrations was the main gelling agent used for the preparation of voriconazole in situ ophthalmic gels. In the drug permeation study, the formulation with the same concentration of PF-68 and the highest concentration of sodium alginate at 1.5% showed the lowest permeation rate after 1 h, and a similar release pattern was observed after 10 h in comparison with 0.5% and 1% of sodium alginate. Furthermore, 16% of PF-68 caused a decline in the drug release rate as opposed to 14% and 15% of PF-68. Moreover, the most sustained release properties were seen at the combination of the highest concentration of sodium alginate, which was 1.5%, with the highest concentration of PF-68, which was 16%. When a higher percentage of each different polymer was used, more entangled polymeric networks were established. Henceforth, the viscosity of the gel increases and forms a more rigid barrier to delay the release and permeation of the drug. The microbiological assay studies manifested that HP-*β*-CD-based Voriconazole in situ ocular gel formulation restrained the growth of *Candida albicans* and *Aspergillus fumigatus* successfully. On the other hand, the inhibition of fungal growth was unsuccessful in the first control group, which is an in situ gel formulation without voriconazole, and control group 2 without the incorporation of a solubility enhancing agent. Therefore, it can be summarized as the incorporation of a solubility enhancer significantly enhancing the antifungal properties of voriconazole in an in situ gel formulation. In addition, the diameter of the zone of inhibition expanded as a greater amount of voriconazole diffused out of the in situ ophthalmic gel [101].

#### 3.4.4. Ocular Viral Infections

Aside from ocular bacterial and fungal infections, the ocular viral infection is a significant cause of ocular morbidity and is very contagious. Antivirals in the formulation of eye drops, eye gels, and eye ointments are commonly given to patients to treat ocular viral infections. Eye drop formulation is the favorite choice of the patients because is it very convenient and easy to be administered. However, low ocular bioavailability is one of the considerable drawbacks as it affects the therapeutic effectiveness. It may also result in unpleasant side effects if high doses are used to improve bioavailability. To resolve this issue, a research article regarding the treatment of viral eye infection using acyclovir with the application of a dual mechanism in situ gelling system written by Ranyadevi et al. was published. In this study, the comparison of a nanoparticle formulation and nanoparticles incorporated into a combination of Pluronic F-127 with Carbopol in situ gel system was made using the dialysis membrane method. A percentage of 15% to 40% of the total loaded acyclovir was released within 1 h after administration; subsequently, an increase in drug release was measured between 60% and 100% within 8 h from the nanoparticle formulation. The nanoparticle formulation had successfully controlled and delayed the acyclovir release for more than 8 h. The release of drug particles within the 1st h may be because some drug particles were not successfully entrapped into the nanoparticle, causing a non-sustained drug release. More drug release occurred later because a greater amount of drug particle was liberated from the nanoparticle. As for nanoparticles incorporated into an in situ gel formulation, 2.75% to 4.22% of acyclovir was released within 1 h after administration and then extended to the maximum level of 6.96% to 32.31% when released after 8 h. The conclusion of these findings was that a more and sustained release was seen in the nanoparticles incorporated in an in situ gel formulation than the formulation with nanoparticles alone. This can be explained by the drug particles that failed to be entrapped in nanoparticles still being entrapped in the in situ gels dispersion media to sustain its release [102].

#### 3.4.5. Other Ocular Diseases

Allergic conjunctivitis is a hypersensitivity reaction of the conjunctiva triggered by many types of allergens. It is very common among patients with manifestations of other allergic problems such as hay fever, asthma, and eczema. This allergic reaction is not contagious and cannot be transmitted to another person. People with allergic conjunctivitis experience signs and symptoms of severe itching, hyperemia, and lacrimation on the eye [110]. Although it is not a life-threatening condition, it causes extreme discomfort to the patient and to some extent affects the daily activities of the patient. Therefore, treatment needs to be given to patients, and researchers had found ways to improve the clinical outcome with the dual mechanism in situ ophthalmic gels.

Ranch et al. stated that the in vivo release of olopatadine HCI was delayed using the combination of pH and ion-activated ophthalmic polymer of Carbopol and gellan gum. The C_max_ of the eye drop group after 5 min of instillation was 13,054 ± 2742 ng/mL, and no drug was measured at 1 h after instillation. In contrast with that, the optimized in situ gel showed a C_max_ of 11,740 ± 1457 ng/mL at 5 min, followed by a sustained release for 3 h. The main reason was that the combination of Carbopol 934P and gellan gum improves the gelation stiffness at the physiological condition due to a synergistic effect and eventually lengthens the drug diffusion route. Therefore, it increases the difficulties of the drug moving across the vicious gel layer onto the surface of the cornea. A more viscous ocular formulation also increases drug retention on the cornea at the same time. Other than that, the permeability coefficient value of the in situ ocular formulation was 1.36 × 10^−5^ sq.cm/s, which was within the desired value range between 1.25 and 1.45 × 10^−5^ sq.cm/s. Benzododecenium bromide (BDB) played an important role in improving the permeability of olopatadine HCL into the site of action. The cationic surfactant property of the BDB improves the corneal permeability of olopatadine HCL by creating pores on the corneal surface. Thus, BDB not only serves as a preservative but also has permeation-enhancing properties in the formulation. The ocular irritation assay showed no symptoms of irritation in the rabbit eyes after applying pH and ion-sensitive-based olopatadine ophthalmic gel. As for the histopathology study of the rabbit cornea after 7 days of treatment, there was no damage in the epithelium layer and no significant histopathological changes in the basal membrane and outermost part of the sub-mucosa compared to the saline solution group. Injuries on the corneal epithelium were not seen after the permeation study of the in situ gel containing olopatadine was carried out. Therefore, the in situ gel formulation is generally safe for the ocular route of administration [103].

Generally, eye irritation is the feeling of dryness, itchiness, pain, or grittiness in the eye. This eye condition can be caused by various reasons, such as blocked tear ducts, dry eyes, infection, as well as irritants. One of the irritants that cause burning, pain sensation, hyperemia, photophobia, and lacrimation on the eye is capsaicin. Capsaicin is an inflammatory compound and is the main ingredient in pepper spray used in policing, riot control, crowd control, and self-defense, including defense against animals. Capsazepine serves as a competitive antagonist of capsaicin on the transient receptor potential cation channel subfamily V member 1 (TRPV1) receptor or known as the capsaicin receptor. In short, capsazepine is analogue and competitive antagonist for the actions of capsaicin. Low bioavailability occurs using conventional eye drop solution of capsazepine due to the nasolacrimal drainage [104].

To resolve this problem, Krishnatreyya et al. developed an in situ ophthalmic gel formulation with the combination of thermosensitive and pH-sensitive in situ gel. Pluronic F-127 functioned as a thermosensitive gelling agent whereas chitosan was a pH-sensitive gelling agent. Capsazepine from in situ gel formulation had only 1.281 ppm recorded in the ocular tissues for the first 0 min, further declined, and was found to be 0.719, 0.530, and 0.470 ppm in the next 10, 15, and 30 min, respectively. No significant amount of capsazepine was detected after 30 min. The outcome indicated that capsazepine in situ ocular gel did not remain in the mammalian eye and was eliminated in a short time. This is favorable in the treatment of eye irritation, which focuses on the surface of the eye and not the interior section of the eye. In the ex vivo permeation study, bovine eyes were used to determine capsazepine permeation between ocular DMSO solution and in situ gel ocular formulation. The maximum permeation of capsazepine in the in situ gel formulation was 97.7% in 12 h, which was slightly lower than capsazepine in the DMSO solution of 98% in 12 h. The apparent permeability coefficient (Papp) for capsazepine in situ gel was recorded as 0.0011 ± 0.0043 cm/s, whereas the Papp for capsazepine solution was 0.0021 ± 0.0036 cm/s. It was interpreted as capsazepine having slower ocular drug diffusion across the in situ gel due to the high viscosity compared to the DMSO solution. In the in vivo efficacy test, *Oryctolagus cuniculus* rabbits were divided into 4 groups: the rabbit eye in group i was the control group, group ii was instilled with capsaicin only, group iii was instilled with capsazepine in situ gel only, and group iv was given capsazepine in situ gel followed by capsaicin exposure. The observation in corneal fluorescein staining revealed that there are no symptoms of irritation, and minimal fluorescein uptake was observed in rabbit eyes in group i and iii. However, obvious signs of irritation and prominent fluorescein uptake were seen in group ii, but it was not subsided within 24 h. Very mild irritation such as freckle-like spot formation and no fluorescein uptake was seen in group iv, which then subsided within 24 h. Hence, the irritation and inflammatory symptoms caused by capsaicin were successfully minimized using capsazepine in situ gel prior to capsaicin exposure [104].

The image of fundus photography using *Oryctolagus cuniculus* rabbits’ eyes showed undamaged retinal blood vessels, and the vitreous media remained optically transparent and translucent without retinal detachments. There was no mechanical injury in the fundus area induced by capsaicin and capsazepine. This indicated that there were no signs of inflammation in the animal fundi. The histopathology studies using *Rattus norvegicus* rats’ eye manifested in an undamaged cornea, and no inflammatory symptoms in the retina were observed. However, minor injury of the epithelia was prominent, and edema and swelling were discovered in group ii. Thus, the results illustrated that the capsazepine in situ gel formulation could be used to treat the inflammation response triggered by capsaicin [104].

Cytotoxicity fluorescent assay was conducted using both the Human Corneal Epithelial Cells (HCEpiC) and Human Retinal Microvascular Endothelial Cells (HRMEC) cultures. Groups i, iii, and iv displayed more noticeable fluorescence patterns (live cells), manifesting that more amount of live cells were present and there was good cytocompatibility between the formulations administered with the ocular cells. In opposition, the number of living cells in group ii was reduced. This was believed that the pro-inflammatory characteristics of capsaicin were the main culprit. Group iv emitted more fluorescence compared to group ii, illustrating the earlier exposure of capsazepine in situ ocular gel before the administration of capsaicin significantly improves cell viability. There was no red fluorescence seen in both media, thereby manifesting that all of the treatment groups did not cause cell mortality. Along with that, a declining trend of cell viability was seen with increasing concentrations of the ophthalmic capsazepine in situ gel compared to the control group for both cultures. This can be explained that although capsazepine is non-cytotoxic, harmful effects may appear if the administered dose exceeds the optimum dose. In conclusion, capsazepine in situ gels could be considered appropriate for ocular application at an optimum concentration [104].

## 4. Expert Opinion

### 4.1. Expert Opinion on Thermo-Responsive In Situ Gelling Systems for Ocular Delivery

Thermo-responsive in situ gelling systems have been proven to be important in designing ophthalmic formulations. This system, in essence, can enhance ocular bioavailability and therapeutic outcomes by prolonging the residence time of therapeutic agents on ocular surfaces (Table 5). Taking that into account, polymers play an immense role. The type of polymers used and their concentration are crucial as they can affect the gel characteristics. Thus, the concentration of the polymers can be finely tuned to achieve desired gelling properties depending on the desired application. As discussed, the most used thermosensitive polymer for ocular drug delivery is poloxamer. According to the studies reviewed, poloxamer showed good drug release properties, low toxicity, and was compatible with other excipients and therapeutic agents. Cellulose derivatives such as HPMC are commonly used as viscosity enhancers in combination with other polymers such as poloxamer. As mentioned, PNIPAAM offers advantages such as good aqueous solubility and lower critical solution temperature (LCST) close to human body temperature. However, it has potential demerits such as low biodegradability and low drug loading capacity, which could be the reasons behind PNIPAAM not being used commonly. The integration of sophisticated drug delivery systems such as nanotechnology with thermo-responsive in situ gelling systems appears to be a judicious choice in improving treatment outcomes. In many cases, the thermally responsive in situ approach is developed for glaucoma and bacterial ocular infections. On the contrary, the research attention for HSK using this approach is lacking. As discussed in the introductory part, the conventional formulation possessed many disadvantages. Still, they are widely used in the treatment of various ocular diseases while there are hardly any thermo-responsive in situ gel products available in the market. Importantly, further investigations will be necessary to support and validate the current research of this approach for the clinician to recognize as well as for future application in HSK or any other ocular diseases therapy.

### 4.2. Expert Opinion on pH-Responsive In Situ Gelling Systems for Ocular Delivery

The main goal of advancing ocular drug delivery is to overcome the problems encountered with conventional drug delivery systems currently available in the market. pH-responsive in situ gelling systems integrate polymers that are capable of responding to the external pH environment, transforming from a solution to a gel matrix. To this present, the nanocarrier is the center of attention with its attractive features. With that, researchers have further stepped ahead by merging nanocarriers together with the in situ gelling systems and disclosed that this strategy further improves sustained release, the prolonged therapeutic action of the drug, enhanced bioavailability, bio-adhesiveness on the ocular surface, better drug solubility, and stability. These characteristics are essential keys in overcoming the problems that present in the conventional dosage form. The integration of such showed to be compatible with a minor alteration of the integrity of the nanocarrier particle [67,68]. In formulating a pH-responsive in situ gelling system, an upward trajectory of carbomer has been observed as the preference polymer mainly due to its ability to gel in a short period and the mucoadhesive property on the mucin layer, which prolonged the formulation residence time on the ocular surface. Although higher concentration produces a better gelling effect, the excessive concentration of carbomer may acidify the formulation, which is further clarified in the safety concern section. PCP also provides a more sumptuous bio-adhesion feature than carbomer but to date has not been fully utilized [111]. Researchers have been paying attention to bacterial conjunctivitis but not herpes keratitis and other ocular diseases in applying this formulation. The challenges in formulating an ideal in situ ophthalmic gel involving several factors should not be overlooked. The overall formulation should have pseudoplastic behavior. It should be fluid enough in the non-physiological environment for the ease of instillation but convert to a gel state and adhere to the ocular surface upon contact with the physiological pH of the eye. The gelation ability should happen immediately to prevent the solution leak during instillation and retain on the ocular surface, resisting the blinking forces and ocular movement. Moreover, with the ability to remain on the eye, the formulation should release the drug in a sustained manner, diffuse from the polymer matrix, and erode slowly during reflex blinking of the eye. In addition, the in situ gel formulation should be stable for an extended period for storage purposes. Safety upon application is important too for long-term and repeated usage of the medication.

### 4.3. Expert Opinion on Ion-Responsive In Situ Gelling Systems for Ocular Delivery

From the current review, we can see that ion-responsive in situ gelling systems have been extensively explored for overcoming the obstacles associated with conventional ophthalmic dosage forms. The selection of the gel base, by and large, depends on its intrinsic properties and envisaged therapeutic use (Table 5). For example, as carrageenan has been proposed to exhibit antiviral activity, its usage in acyclovir in situ gel to treat herpes keratitis would obviously be a wise decision, as this can provide a desirable synergistic therapeutic outcome. The percentage of gel base influences gelling strength, which in turn affects the drug release rate. Although gellan gum is the most common agent used, it can be combined with other ion-sensitive polymers, such as sodium alginate or carrageenan, to improve gel strength, mucoadhesion, residence time, and drug release profile. Other studies have found that the combination of alginate with poloxamer (thermo-responsive in situ polymer) can reduce the phase transition temperature and allow less poloxamer concentration to be used. Furthermore, blends of nanotechnology and in situ gelling systems (topical nanogels) for ocular delivery are promising fruition for effective and sustained drug delivery to the posterior segment of the eye, to replace current invasive approaches such as intravitreal injection or systemic oral or injection dosage, which are associated with more adverse reactions. Overall, as far as efficacy is concerned, the ion-responsive in situ ocular systems appears to be more superior over conventional eye drops in aspects such as prolonged ocular contact time, sustained drug delivery, and ocular bioavailability. Recent applications of ion-sensitive in situ gels have been focusing on glaucoma, bacterial, and fungal eye infections, as evidenced by an impressive number of research articles available. Hence, we are confident that an in situ gelling system could be a new paradigm for treating HSK. Nevertheless, there are some limitations associated with the research studies mentioned above. For instance, the types of in vitro, in vivo, and ex vivo assays are not standardized across all studies. Some experiments do not provide ocular disposition data, whereas others are lacking ocular permeation and pharmacodynamic studies. Therefore, a consensus among formulators with respect to the specific assays required may be necessary for quality evaluation of future formulations.

### 4.4. Expert Opinion on Multi-Stimuli-Responsive In Situ Gelling Systems for Ocular Delivery

We agreed that in situ gel formulation, to a great extent, improved active drug delivery outcomes to the administration sites. Sadly, the physiological condition of the ocular site is very complicated. Therefore, multi-stimuli-responsive in situ gel formulations which enable more accurate adjustment through more parameters had been developed to achieve better ocular drug delivery outcomes in such complex physiological conditions. Most of the multi-stimuli responsive in situ gel formulations have outstanding sustained drug release properties, while some have excellent control drug release properties compared to eye drop solution, suspension, and single mechanism in situ gel formulation in the treatment of many ocular diseases. In addition, more sustained drug release properties were observed as the concentration of the polymer increases. Research studies had achieved higher or desired ocular bioavailability and an ocular permeation rate using multi-stimuli-responsive in situ gel formulations compared to eye drop solution, suspension, and single mechanism in situ gel formulation. The anti-microbial efficacy study of besifloxacin in *Pseudomonas aeruginosa* and *Staphylococcus aureus* with the instillation of in situ gel is better than the suspension formulation using the agar diffusion test method.

Among all of the ocular diseases discussed above, bacterial ocular infections received the most attention in the research study of dual and tri-mechanism in situ ocular gel recently. There are a total of four research studies discussed above such as the treatment of bacterial ocular infections using sparfloxacin, the treatment of bacterial conjunctivitis with the use of besifloxacin, the treatment of bacterial keratitis using levofloxacin, as well as the treatment of bacterial endophthalmitis by applying chloramphenicol and dexamethasone sodium phosphate. Second to 4th generation quinolones are very popular as topical antibiotics in ocular infection because of their extensive coverage for possible causative bacteria such as Gram-positive, Gram-negative, and atypical bacteria [112].

The combination of pH and ion-sensitive in situ gel formulation is mostly used in the treatment of ocular disease. The reason is believed to be that the gelation of pH and ion-sensitive polymer happens immediately upon pH environment changes to 7.4. The time of ion-sensitive gelation also happens as soon as it is stimulated by the changes of ion. On the other hand, the thermally responsive polymer has a slow temperature response, required very high polymer concentration in preparation, and has low mechanical properties. Therefore, pH and ion-responsive polymers are more preferred to be used in combination for ocular drug delivery in recent studies [113,114].

## 5. Safety Concern

### 5.1. Safety Concern Related to Thermo-Responsive In Situ Gel

**Figure 8:**
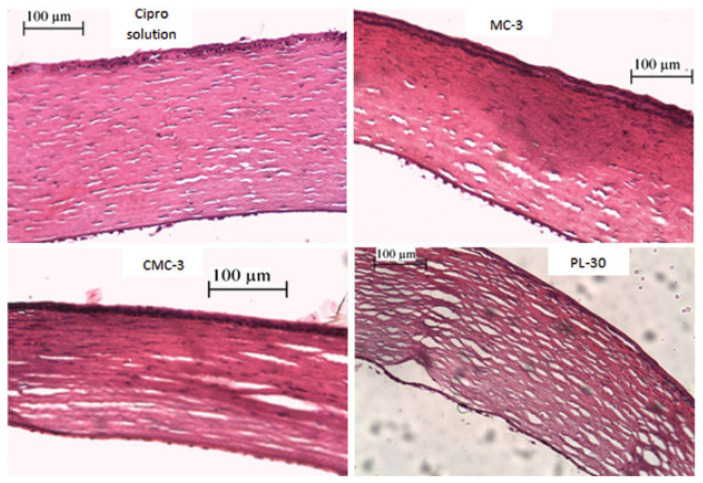
Photomicrographs of corneal after the administration of ciprofloxacin solution, methylcellulose (MC)-based preformed gel, CMC pre-formed gel and Pluronic F-127-based in situ gel [32].

The safety profile is important to take in consideration in order to develop a safe and efficient thermo-responsive ophthalmic in situ gel. Based on the studies discussed above, the use of thermosensitive polymers is generally safe without causing any irritation in animal models. However, a study conducted by Abdelkader and Mansour [32], as discussed previously, has reported negative effects of the thermosensitive polymers (Figure 8). The authors compared the histological examination of Pluronic F-127-based thermosensitive in situ gel with cellulose pre-formed gel for the treatment of bacterial keratitis. The results revealed that the cellulose-based gel showed better ocular tolerability as it caused only mild stromal edema. In contrast, the Pluronic-based gel increased the stromal thickness by around 1.5-fold more than cellulose-based gel. The study suggested that this can be due to the high concentration of Pluronic used in this formulation, which is 30% [32].

Apart from that, Güven et al. [50] developed a thermosensitive in situ gel loaded with olopatadine hydrochloride for ocular allergy. The polymers used in this study were Pluronic F-127 and HPMC. The cytotoxic evaluation disclosed that Pluronic F-127 was more cytotoxic compared to HPMC. However, the study suggested the cytotoxicity only occurred at high doses indicating that the cytotoxicity is concentration-dependent, which was in line with the study by Abdelkader and Mansour [32]. The concentration of Pluronic used in the previous studies reviewed did not exceed 30%; this may be the reason why minimal or no ocular irritation was observed. According to the studies reviewed, the addition of other types of polymers such as HPMC can decrease the concentration of Pluronic used without compromising the gelling characteristics.

### 5.2. Safety Concern Related to pH-Responsive In Situ Gel

The safety of carbomer has been well established back in the 1990s, including carbomer-910, -934, -941, and -940 [115]. According to the literature discussed above, researchers predominantly used carbomer-934, -940, and -974, whilst only one study utilized carbomer 943. The safety of PCP was evaluated by Krenzer et al. by regularly administering it to the NZW rabbits’ eye with a corneal epithelial defect, penetrating corneal incision, or laser in situ keratomileuses (LASIK) flap for one year [116]. The results showed no difference between PCP-treated corneas and controls, which indicates that PCP is safe in long-term usage.

Most of the developed pH-responsive in situ gelling systems discussed above were proven to be biocompatible and safe to use in the ocular region. Among the literature covered, two studies were reported to have mild irritation. Jaiswal et al. formulated a polymeric micellar in situ gel system of itraconazole and observed discoloration in two eggs at time points of 480 min and 720 min [69]. A chitosan nanoparticulate in situ gelling system formulated by Upadhayay et al. also revealed that the 12th h of the HET-CAM test showed visible membrane discoloration but without hemorrhage, suggesting mild irritation [68]. Histological examination on goat cornea further carried out displayed that the epithelial layers of the corneal were intact. Both studies happen to have not incorporated HPMC or MC during the formulation as other studies did. The addition of HPMC or MC was believed to lower down the acidity of the carbomer polymer without affecting its gelling ability. It also acts as a viscosity enhancer, strengthening the gel structure resisting blinking forces and eyeball movement. From the author’s point of view, these mild irritations are mainly attributed to the sudden shift of pH of the acidic formulation to neutral pH in the presence of carbomer. A higher amount of carbomer is often incorporated to achieve excellent gelling capacity, which ultimately acidifies the formulation. Therefore, HPMC is typically added in the formulation with a higher portion than carbomer to lower the concentration of carbomer needed while serving as a viscosity enhancer. In addition, the viscosity of the overall formulation should not be too high nor too low. Extreme viscosity with poor pseudoplastic may irritate the eyes due to the high stiffness of the gel. At the same time, too low viscosity failed to achieve gelation and mucoadhesion on the ocular surface, making it impossible for sustained release [64,66]. Furthermore, the formulation should not affect the hydration level on the ocular surface when the gel swells. Noreen et al. highlighted that the formulated *T. arjuna* gum can retain the corneal hydration level within 75–80%, thereby not harming the corneal tissues [66].

### 5.3. Safety Concern Related to Ion-Responsive In Situ Gel

Gellan gum, alginic acid, and carrageenans are usually selected as ion-responsive polymers used for ocular delivery. Previously, carrageenan and gellan gum are FDA-approved safe for use as food additives, while alginates are FDA approved for dressing and food additives. The majority of the studies reviewed showed that the in situ ocular gelling systems based on gellan gum, alginates, and carrageenan are well tolerated when tested on animals. As an example, Fernandez-Ferreiro et al. developed the ion-sensitive hydrogels containing trypan blue, which are based on all gellan gum, kappa-carrageenan, and alginates for ophthalmic delivery [117]. To assess the ocular safety, the ion-sensitive hydrogel was instilled into the eye of male Sprague–Dawley rats every 12 h for 3 months. The signs of anterior surface damage, conjunctival injury, redness, or increased tearing were not detected by direct visual observation or immunohistochemical techniques. Despite this, Li et al. reported some incidences of ocular toxicities associated with permeation enhancers [93]. In particular, EDTA, F68, and Azone resulted in more than 83% corneal hydration level compared to the standard level (76–80%). Moreover, they also increased the frequency of eyelid blinking. Hence, permeation enhancers must be used with caution to avoid harmful effects to the eyes. Sometimes, nanoformulation may experience an initial high burst, releasing drug amounts that can be toxic. However, this issue may be alleviated when combined with in situ gel, as the gelation network has been shown to prevent initial drug burst release and allow controlled release for a longer time. Nevertheless, the stability of natural polymers such as gellan gum, alginate, and carrageenan remains another area requiring longer speculation to ensure ocular safety. The degradation of alginate is observed due to an extreme pH environment. For instance, H^+^-catalyzed hydrolysis of alginate is predominant at pH < 5, whereas degradation via β-alkoxy-elimination is observed at pH > 10. Moreover, the presence of enzymes may also affect the stability of natural polymers. Thus, caution should be taken when formulating in situ gels with natural ionic responsive polymers to avoid toxicity issues. Investigations to discover newer biopolymers with enhanced stability and biocompatibility is highly encouraged.

### 5.4. Safety Concern Related to Multi-Stimuli-Responsive In Situ Gel

The techniques used to examine the safety profile of those polymers include the HET-CAM test, gamma scintigraphy study, handheld infra-red (IR) assay, histopathology study, and in vivo eye irritation assay. In conclusion, most of the polymers in multi-stimuli responsive in situ gel formulations did not cause any signs of eye irritation. There are merely two studies that reported that chitosan and gellan gum-based in situ gel formulation caused mild, tolerable irritation to the eye. According to two research studies carried out by Gupta et al., a modified HET-CAM test was performed on the chitosan and gellan gum-based in situ formulation and normal saline. The observation revealed that normal saline did not cause any irritation to the eye, whereas the innovative formulation caused mild irritation to the eye [96,97].

Some studies also revealed that a multi-stimuli responsive in situ formulation was effective in the prevention of active ingredients from entering into systemic circulation through conjunctival blood capillaries and lymphatics by nasolacrimal drainage. A gamma scintigraphy study conducted by Gupta et al. shows that timolol maleate, sparfloxacin, and levofloxacin from plain ocular solution was rapidly removed by nasolacrimal drainage and some of the timolol maleate, sparfloxacin, and levofloxacin were detected in the kidney and bladder of the rabbits. Conversely, timolol maleate, sparfloxacin, and levofloxacin from innovative in situ ocular gel formulation has relatively longer retention on the corneal surface at a longer period in comparison to plain ocular drug solution and was not detected in the kidney and bladder of the rabbits [96,97,99].

In summary, the multi-stimuli responsive in situ gel formulation is regarded as a safe formulation for the management of various ocular diseases.

## 6. Clinical and Safety Aspect

### 6.1. Clinical and Safety Aspect Related to Thermo-Responsive In Situ Gel for Ocular Delivery

The thermosensitive ophthalmic gel was developed by Akorn Inc. Pharmaceuticals under the trade name Akten^®^ received approval from the FDA in 2008 [6,118]. Akten^®^ is a preservative-free lidocaine (3.5%) ocular anaesthetic indicated for use in ocular procedures with the dose of two drops applied to the ocular surface. The formulation contains lidocaine hydrochloride, HPMC, sodium chloride, and water for injection [119]. Owing to the presence of HPMC, the contact time between the formulation the corneal and conjunctival can be increased. The appropriate viscosity of this formulation can reduce systemic absorption and thus the possible systemic side effects. Moreover, the viscosity of the formulation was 50% less compared to the gel for non-ophthalmic preparations for ease of administration [118]. The clinical study of this product was conducted as a multi-center, randomized, controlled, and double-blind study to evaluate the effectiveness and safety of Akten^®^ in 209 subjects. The subjects were divided into four cohorts where they were randomized to the sham, Akten^®^ 1.5%, Akten^®^ 2.5%, and Akten^®^ 3.5%, respectively. The conjunctiva of each subject was pinched using a forcep to determined the anesthetic effect. Particularly, 92% of the subjects from the Akten^®^ 3.5% group achieved anesthesia within 5 min upon the application of the formulation. In this group, about 84%, 55%, and 27% of the subjects experienced anesthesia for at least 5 min, more than 10 min, and more than 15 min, respectively. The clinical study concluded that Akten^®^ is effective and safe to be used as an ocular anesthetic [118,119]. However, it is important to mention that long-term use of ocular anesthetic may cause ulceration and opacification of the corneal owing to delayed wound healing [118,119]. The commonly reported side effects include burning sensation, headache, conjunctival hyperemia, and corneal epithelial changes [119].

### 6.2. Clinical and Safety Aspect Related to pH-Responsive In Situ Gel for Ocular Delivery

Currently, there are no ongoing clinical trials on pH-responsive in situ gel for ocular delivery. Previously, Sirion Therapeutics Inc. formulated pH-responsive in situ ophthalmic gel containing ganciclovir 0.15% as the main active ingredient targeting HSK. The formulation was approved by the FDA in 2009 with the trademark Zirgan^®^ [120]. Ganciclovir was formulated with carbomer 974P, water for injection, sodium hydroxide, mannitol, and a proper amount of benzalkonium chloride [120]. There was a total of four clinical trials conducted. Firstly, comparing the healing rate of acyclovir 3% ophthalmic ointment with ganciclovir 0.15% gel in a sample size of 164 with HSK condition in one open-label, randomized, controlled, and multi-center trial revealed no substantial difference. Another three clinical trials with the same purpose carried out in a sample size of 213 that were single-blinded, randomized, controlled, and multi-center also revealed no difference in healing rate [120,121]. The optimal dose in these trials was five times daily with one-drop instillation until fully healed, which was followed by three times daily for a week. During the trials, the adverse complaint reported included blurred vision, ocular irritation, punctate keratitis, and conjunctival hyperemia but were all reported to be low [121,122]. Post-marketing surveillance revealed that occasional eye irritation such as mild burning, tingling sensation, or blurred vision was reported to be rare and does not mandate withdrawal from the market. In addition, another trial carried out in Chinese patients comparing the effectiveness and safety of ganciclovir 0.15% pre-formed gel with in situ gel also disclosed that both were equally effective and in situ gel has better ocular tolerance than pre-formed gel [123].

### 6.3. Clinical and Safety Aspect Related to Ion-Responsive In Situ Gel for Ocular Delivery

The clinical trial was conducted to evaluate the efficacy and safety of the long-term application of carteolol alginate gel-forming solution compared with standard carteolol solution [124]. The trial was based on a double-masked, parallel group, and the trial was multi-center. Patients with open angle glaucoma or ocular hypertension were randomly distributed to receive carteolol alginate or standard solution, and treatment efficacy was evaluated at baseline, 15, 60, and 120 days, respectively. The clinical trial results found that the carteolol alginate given once daily was equivalent in ocular hypotensive efficacy to the standard solution given twice a day, with an approximate 25% reduction in IOP [124]. Moreover, the ocular tolerance of carteolol alginate gel was reported to be as good as the standard formulation, highlighting the non-irritant feature of the alginate base [124]. As such, the clinical trial successfully concluded that the new carteolol alginate formulation was as effective and safe as the standard carteolol solution.

### 6.4. Clinical and Safety Aspect Related to Multi-Stimuli-Responsive In Situ Gel for Ocular Delivery

A randomized, double-blinded, vehicle-controlled, parallel-group clinical trial conducted by Hosseini et al. to investigate the efficacy, safety, and tolerability of 0.075% bromfenac ophthalmic in situ solution with the brand name of BromSite^®^ [125]. BromSite^®^ was approved by the US FDA for the management of postoperative inflammation of pain in patients after cataract surgery in 2016. Bromsite^®^ is marketed by Sun Ophthalmics and poloxamer 407 and polycarbophil were used as an in situ gelling polymer [126]. The comparison was made between the bromfenac in situ gel formulation and in situ gel formulation alone. The first dose was administered to the volunteers on day 0, which was one day before the cataract surgery took place. After the completion of the surgery, the second dose was administered in the evening. Two daily doses were continued for 2 weeks after the cataract surgery. The outcome showed that 76.8% of the subjects that received 0.075% Bromfenac in situ ocular formulation had a 0 pain score on the day after the cataract surgery, whereas 48.2% of the subjects that received the in situ gel alone had a 0 pain score on the day after the cataract surgery. The percentage of the subjects that had a 0 pain score was significantly higher in the 0.075% Bromfenac formulation group than in the group with in situ gel alone on the following visits on day 8, 15, and 29. This outcome proved that the Bromfenac in situ ocular formulation remarkably relieved the postoperative inflammation of pain in patients after cataract surgery. Other than that, some subjects experienced adverse effects in the middle of the clinical trial such as eye pain, iritis, ocular hypertension, and headache. Most of the adverse effects were in mild to moderate condition, and only a few subjects encountered these adverse effects [125]. Therefore, this clinical trial had concluded that Bromfenac in situ ocular formulation is an appropriate topical medicament for the alleviation of postoperative inflammation of pain in patients after cataract surgery.

## 7. Conclusions and Future Directions

The eye is the primary sensory organ for vision, acting as the window to the world. Different structures within the eyes must function closely in harmony with one another, allowing humans to capture nature’s beauty and build connections with their surroundings. However, as the eyes are exposed directly to the external environment, they are susceptible to the vicissitudes of diseases. Therefore, prompt treatment is typically given to all patients to relieve symptoms and prevent worsening of the condition. Topical treatment remains the preferred choice owing to the ease of administration as well as less invasive and affordable cost. Unfortunately, the well-established ocular self-defense mechanism limits the clinical effectiveness of the current topical antiviral formulations. For instance, the blinking reflex and tear turnover give rise to rapid precorneal drug elimination into the nasolacrimal duct, causing undesirable systemic absorption and meager ocular bioavailability. Ointments are better at prolonging the precorneal contact time, but many reported blurred vision and difficulty in administration, leading to poor patient acceptance and compliance. Regardless of the challenges in ophthalmic preparations, numerous novel approaches have been developed to obviate the obstacles faced by conventional ocular formulations over the years. Undeniably, the development of in situ gel-forming solutions is one of the best, applaudable research outcomes. In brief, thermo-responsive, pH-responsive, and ion-responsive in situ gels individually possess good gelling ability, which are designed for treating and preventing various ocular diseases. In fact, it was found that the multi-stimuli responsive formulation provides good gelling characteristics with lower polymer concentration, hence the minimal risk of toxicity compared to single stimulus-responsive polymers. Such smart ocular delivery, in essence, boosts the therapeutic outcome by prolonging the drug release and contact time attributed to the sol–gel transformation and mucoadhesive behavior. In addition, the amalgamation of the nanotechnologies, prodrugs, and peptide drug delivery with in situ gelling systems boosts the ocular targeting efficiency. Most of the present studies showed that stimuli-responsive in situ formulations are generally safe, while the minority reported mild irritation, which can be modified to improve ocular tolerance. Therefore, this current review concludes that stimuli-responsive in situ gel is one of the preferred drug delivery systems for treating ocular herpetic infection. Nevertheless, the majority of the studies were conducted preclinically with animal models within a short observation duration. Indubitably, future additional clinical investigations are mandatory to evaluate the reproducibility, safety, and toxicity of the advanced topical formulations to avoid regulatory hurdles during commercialization. Additionally, blends of nanotechnology and in situ gelling systems (topical nanogels) for ocular delivery are promising for effective and sustained drug delivery and must be explored for therapeutic efficacy and safety.

## Figures and Tables

**Table 1:** Smart ocular delivery using thermo-responsive in situ gel approach against ocular diseases.

Objective	Disease/Drug	Types of Stimuli/Polymer Used	Membrane/ Cell Line/ Animal Model	Outcome	Source
To develop thermo-responsive in situ gel for ocular delivery of latanoprost.	Glaucoma/latanoprost	Temperature/Pluronic F-127, Pluronic F-68, HPMC E5, and HPMC E50	New Zealand White (NZW) rabbits	Gelation temperature: 34.3 °C pH: 6.53 Mucoadhesive properties: Mucoadhesion: 0.06 mJ Maximum detachment force: 0.09 N Ex vivo permeation: 53.5 μg/cm^2^ In vivo antiglaucoma efficacy: the optimized formula showed a 2.9-fold higher AUC value than Ioprost eyedrop. In vivo ocular irritation test: no sign of corneal tissue damage.	[24]
To prepare and investigate sustained-release thermo-responsive in situ gel of bimatoprost.	Glaucoma/bimatoprost	Thermo-responsive/ poloxamer 188, poloxamer 407, and HPMC K4M	Chorioallantoic membrane (CAM)	Gelation temperature: 37.5 °C pH: 7.3 ± 0.31 Gelling capacity: +++ (rapid gelation and remained for 10 h) Viscosity: 7.65–82.24 cps (sol) 238.16–2335.52 cps (gel) Rheology: pseudoplastic In vitro release: 80.37–86.63% of drug released for 10 h. Ex vivo permeation: 67.45% of the drug was permeated up to 12 h. In vitro ocular irritation test (HET-CAM): irritation score of 0.04 indicating no irritation.	[25]
To evaluate and compare the effectiveness of betaxolol hydrochloride loaded thermo-responsive in situ gels with ophthalmic resin suspension.	Glaucoma/betaxolol hydrochloride	Thermo-responsive/poloxamer 407 + HPMC (poloxamer-based gel) MC+PEG 4000 (MC-based gel)	Rabbits	Poloxamer-based in situ gel Gelation temperature: 29.16 ± 0.21 °C Gel capacity: 9.48 ± 0.29 min In vitro drug release: Increasing P407 concentration from 18% to 22% decreased the release percentage from 85% to 53% in 6 h. Sustained release In vivo distribution: the AUC and MRT of were 2-fold higher than the commercial resin suspension (Betoptic S^®^ eye drops). MC-based in situ gel Gelation temperature: 34 °C In vitro drug release: increasing PEG4000 concentration from 3% to 7% decreased the release percentage from 68% to 43% in 4 h. Sustained release In vivo distribution: the AUC and MRT of both in situ formulations were 2-fold higher than the commercial resin suspension (Betoptic S^®^ eye drops).	[26]
To prepare thermo-responsive in situ gel to improve the bioavailability of timolol maleate.	Glaucoma/timolol maleate	Thermo-responsive/poloxamer 407 and poloxamer 188	NZW Rabbits	Gelation temperature: 32 °C pH: 6.70 ± 0.10 Rheology: pseudoplastic In vivo pharmacokinetics: MRT of TM in situ gel was 1.6-fold higher than TM eye drops. AUC of TM in situ gel was higher than TM eye drops. In vivo pharmacodynamics: TM loaded in situ gel exhibited a more significant IOP lowering effect compared to TM eye drops. Histopathological study: the formulation was well biocompatible, and no irritation was observed.	[27]
To fabricate controlled drug release thermosensitive in situ gel of ofloxacin.	Bacterial conjunctivitis/ofloxacin	Thermo-responsive/ poloxamer 407 HPMC, and PVA (mucoadhesive polymers)	-	Gelation temperature: <32 °C pH: 6.6–7.2 In vitro drug release: initial burst effect was observed. The prepared formulations demonstrated sustained drug release for 9 h. In vitro antimicrobial studies: all in situ formulations are effective in antimicrobial but the ZOI was smaller compared to the marketed formulation (0.3% *w*/*v* ofloxacin eye drops).	[28]
To formulate levofloxacin hemihydrate loaded ocular in situ gel.	Bacterial conjunctivitis/levofloxacin	Thermo-responsive emulsomal/Pluronic F-127 and Pluronic F-68	Rabbits	Gelation temperature: 32.75 ± 0.35 °C Rheology: non-linear plastic flow In vitro drug release: optimized formulation exhibited prolonged drug release up to 12 h. Optimized formulation exhibited prolonged drug release up to 12 h. No change in release pattern over 3 months. In vivo microbiological susceptibility: optimized formulation Retention time: 12 h Inhibition zone: decreased after 8 h Levoxin^®^ eye drops Retention time: 4 h. No inhibition zone was observed after 4 h. In vivo ocular irritation test: non-irritant.	[29]
To synthesize thermo-responsive emulsomal in situ gel to enhance the therapeutic efficacy of sparfloxacin.	Bacterial conjunctivitis/sparfloxacin	Thermo-responsive/ Pluronic F-127, and Pluronic F-68	Rabbits	Gelation temperature: ≈35 °C pH: 7.4 Gelling capacity: +++ (gelation occurred within 2–3 s and remained for >2 h.) Viscosity: Before gelation (25 ± 1 °C): 107.46 ± 6.74 cps After gelation: (37 ± 1 °C): 1669 ± 13.89 cps In vitro release: 75.274 ± 0.17% of drug released at 12 h. Ex vivo permeation: 66.203 ± 2.39% of the drug was permeated through goat cornea. In vivo antimicrobial efficacy: the symptoms were reduced within 4–5 days with the use of optimized formulation. In vivo ocular irritation test: non-irritant.	[30]
To fabricate and investigate the sustained release in situ gel of ciprofloxacin.	Bacterial keratitis/ciprofloxacin	Thermo-responsive/poloxamer 407 and HPMC	Rabbits	Gelation temperature: 30 ± 0.01 °C pH: 4.57 Gelling capacity: +++ (rapid gelation and remained for an extended period) Rheology: pseudoplastic In vitro drug release: 83% of drug release from the optimized formulation in 8 h while the nearly total amount of drug release from marketed eye drop solution in 10 min. In vivo elimination study: elimination of optimized in situ gel was within 1 h. In vivo ocular irritation (Draize test): non-irritant.	[31]
To compare the characterisation and evaluation of pre-formed gel and in situ gel for delivery of ciprofloxacin.	Bacterial keratitis/ciprofloxacin	Thermo-responsive/MC and CMC (preformed cellulose-based gel) Pluronic F-127 (in situ gel)	Rabbits	Pluronic-based in situ gel Gelation temperature: 35 °C pH: 5.0 Rheology: pseudoplastic In vitro drug release: 20%.h^−0.5^ In vivo study: faster corneal healing was observed (<5 days) compared to ciprofloxacin solution. Histological examination: significant stromal edema MC-based pre-formed gel pH: 5.1 Viscosity: 10,000 mPa.s In vitro drug release: 33%.h^−0.5^ In vivo study: faster corneal healing was observed (<5 days) compared to ciprofloxacin solution. Histological examination: minimal stromal edema CMC-based pre-formed gel pH: 6.5 Viscosity: 8300 mPa.s In vitro drug release: 31%.h^−0.5^ In vivo study: faster corneal healing was observed (<5 days) compared to ciprofloxacin solution. Histological examination: minimal stromal edema	[32]
To fabricate and evaluate voriconazole-loaded thermo-responsive in situ gel.	Fungal keratitis/voriconazole	Thermo-responsive/poloxamer 188, poloxamer 407 and/or CMC	NZW rabbits	Gelation temperature: 34.13 ± 0.32 °C pH: 6.80 ± 0.03 In vitro drug release: 83.5% of drug released from the formulation at 12 h indicating sustained release. Ex vivo permeation: 42.92 ± 6.81% of drug permeated through cornea after 24 h. Voriconazole concentration in tear (in vivo): the drug concentration in tear fluid was higher for optimized in situ gel compared to the solution. In vivo Draize Rabbit Eye test: irritation score <1, non-irritant.	[33]
To synthesize, characterize, and investigate the sustained release in situ formulations of voriconazole.	Fungal keratitis/voriconazole	Thermo-responsive/poloxamer 188, poloxamer 407, and poloxamer 388	-	Gelation temperature: 32.200 ± 0.265 °C pH: 6.357 ± 0.006 Gelling capacity: 1.233 ± 0.058 s Adhesiveness: 0.558 ± 0.005 g⋅s at 25 °C 5.600 ± 1.024 g⋅s at 32 °C Rheology: pseudoplastic In vitro drug release: prolonged drug release up to 24 h.	[34]
To prepare PNIPAAM–hyaluronic acid-based in situ gel to deliver ketoconazole.	Fungal keratitis/ketoconazole (KCL)	Thermo-responsive/PNIPAAM-hyaluronic acid	NZW rabbits	Gelation temperature: 33 °C pH: 6.0–7.5 In vitro drug release: The drug release of in situ gel (30% drug released at 2 h) slower than in free drug (95% drug released at 2 h). In vivo antimicrobial study: 91.7% and 66.7% of cure rate were obtained in ketoconazole in situ gel and commercial ketoconazole eye drops, respectively. In vivo ocular irritation test: irritation score of zero was obtained for developed formulation.	[35]
To develop and evaluate thermo-responsive in situ gel nanoemulsions in delivering acyclovir.	Herpes simplex keratitis/acyclovir (ACV)	Thermo-responsive nanoemulsion/ Triacetin, and Transcutol^®^ P (nanoemulsion) poloxamer 407 and poloxamer 188 (in situ)	NZW rabbits (in vivo ocular irritation test) and CAM (in vitro ocular irritation test)	Gelation temperature: 30.9 °C pH: 4.58 ± 0.068 Viscosity: 103.03 ± 4.68 mPa.s In vitro drug release efficiency: 80.78 ± 1.82% The optimized formulations displayed a sustained release manner. Ex vivo permeation: the permeation of ACV was 2.83-fold higher in optimized formulation compared to ACV solution. In vivo ocular irritation test: minimal conjunctival redness but disappeared after 2 h of administration. In vitro ocular irritation test (HET-CAM): cumulative score of 0.33 ± 0.58 indicating non-irritant	[36]
To evaluate the efficacy of nintedanib thermosensitive hydrogel.	Neovascularization/nintedanib	Thermo-responsive/poloxamer 407	Rat	Gelation temperature: 37 °C In vivo studies: The CNV area of alkali burn rats was significantly reduced from day 3 to day 14. The CNV area was found to be the lowest in NTH group on day 14 compared to model group, dexamethasone group and normal control group. Immunofluorescence: the developed formulation reduced the expression of VEGFR-2 and CD31	[37]
To fabricate and optimize sustained-release thermosensitive in situ formulations of tetrahydrozoline.	Ocular allergy/tetrahydrozoline (THZ)	Thermo-responsive/poloxamer 407 and poloxamer 188	NZW rabbits	Gelation temperature: 31.08 ± 0.34 °C pH: 7.010 ± 0.017 Gelling capacity: 1.9 ± 0.1 s Viscosity: 504.2 ± 3.5 cps In vitro drug release: 88.745 ± 8.275% of THZ release from optimized formulation at the end of 24 h. The sustained release was achieved. Ex vivo permeation: 2.035 ± 0.062% of THZ was permeated. Ex vivo penetration: 0.582 ± 0.035% of THZ was detected in corneal tissue. In vivo studies: the concentration of THZ (1.029 μg/mL) in tear sample was higher in optimized formulation compared to THZ marketed product (no drug detected) at 6 h. In vivo ocular irritation: non-irritant	[38]
To develop disulfiram solid dispersion thermosensitive in situ gel to enhance corneal permeability and aqueous solubility.	Cataract/disulfiram	Thermo-responsive/poloxamer 407 and poloxamer 188	NZW rabbits	In vitro corneal permeation: the formulation with the ratio of 1:5 (DSF to poloxamer 188) showed 2.31-fold higher apparent permeability coefficient than DSF eye drops. In vivo precorneal retention study: the formulation successfully prolonged the retention of fluorescence for 30 min. In vivo efficacy: thermosensitive in situ formulation had better anti-cataract efficacy among blank group, model group, DSF suspensions group, and DSF_SD_ suspensions group at day 7 post-administration. In vivo ocular irritation: non-irritant.	[39]

**Table 2:** Smart ocular delivery using pH-responsive in situ gel approach against ocular diseases.

Objective	Disease/Drug	Types of Stimuli/Polymer Used	Membrane/ Cell Line/ Animal Model	Outcome	Source
To develop in situ gel formulation of timolol maleate based on gelling properties of Carbopol and chitosan combination and evaluate reduction of IOP compared with the liposomal and marketed formulation.	Glaucoma/Timolol maleate	pH-responsive/Carbopol and Chitosan	-	pH: 6.00 Gelation pH: 7.4 (STF) Gelation time: +++ (gel immediately and remained extended period) Viscosity: 1040.00 cps (gel) Rheology: pseudoplastic flow In vitro release: cumulative release 60.9% up to 24 h. In vivo distribution: Developed in situ gel showed a 2.4-fold greater AUC compared to marketed eye drops. Developed in situ gel showed a 2.1-fold greater AUC compared to the liposomal formulation.	[56]
To investigate the potential of pH-responsive hydrogel containing a combination of timolol maleate and brimonidine tartrate as an ocular drug delivery system to treat glaucoma.	Glaucoma/Timolol Maleate and Brimonidine tartarate	pH-responsive/Carbopol 934P and HPMC	White rabbits	Gelation pH: 7.4 (phosphate buffer) Viscosity: 33.2 cps (pH 4) and 56.3 cps (pH 7.4) In vitro release: cumulative released ≈80% of combination drug up to 8 h. In vivo efficacy: demonstrate 7 h longer IOP reduction compared to marketed eye drops. In vivo ocular irritation study: no sign of corneal damage.	[57]
To develop in situ gelling of brimonidine tartrate with a lower concentration (0.05%, 0.1%, and 0.2% *w*/*v*) and evaluate its efficacy with eye drops (0.2% *w*/*v*) and reduction of systemic absorption.	Glaucoma/Brimonidine tartarate	pH-responsive/Carbopol 974P and HPMC E4M	New Zealand White (NZW) rabbits	pH: 5.93–6.07 Residence time: up to 3 h In vivo efficacy: The lowest concentration (0.05%) showed 1.2 times better IOP reduction compared to 0.2% eye drops. All in situ gel formulation showed low systemic absorption, suggesting fewer systemic side effects. In vivo ocular irritation study: No sign of corneal damage. Histological examination demonstrated that epithelial remained intact.	[58]
To evaluate ex vivo and in vivo performance of brimonidine tartrate in situ gel as compared to marketed eye drops.	Glaucoma/Brimonidine tartarate	pH-responsive/Carbopol 974P and HPMC K4M	-	Gelation pH: 7.4 (artificial tears fluid) Gelation time: +++ (gel immediately and remained extended period) Viscosity: 4062 ± 138 cps (gel) In vitro release: cumulative released 90.77% up to 8 h. Ex vivo permeation: Permeability coefficient (P): $28.25\;\pm 1.18\;\times \;10^{3}\;\mathrm{cm}/h$ Permeation flux (J): $28.25\;\pm 1.1\;\mu g/{\mathrm{cm}}^{2}h$ 76.83% of brimonidine was permeated up to 5 h. In vivo efficacy: demonstrated 3.4 times better IOP reduction and 5 h longer than marketed eye drops.	[59]
To develop an in situ gel system that can reside for a long time and prolong the drug release by using Carbopol 940 and HPMC.	Glaucoma/Brimonidine tartarate	pH-responsive/Carbopol 940 and HPMC	NZW rabbits	pH: 4.2 Gelation pH: 6.7 (artificial tears fluids) Gelation time: +++ (gel immediately and remained extended period) Viscosity: 754 ± 0.01 cps (gel) In vitro release: cumulative released 87.46 ± 0.15% up to 8 h. In vivo ocular irritation study: no sign of corneal damage.	[60]
To develop pH-responsive in situ gel for ophthalmic delivery of dorzolamide hydrochloride by using Carbopol.	Glaucoma/Dorzolamide hydrochloride	pH-responsive/Carbopol 940 and HPMC F4M	-	pH: 5.16 ± 0.01 Gelation pH: 7.4 (STF) Viscosity: 5.531 p (sol) and 18.374 p (gel) Rheology: pseudoplastic flow Mucoadhesion force: 0.3346 ± 0.001 nm (positive absorbance indicates interaction between formulation with mucin fluid) In vitro release: sustained release up to 24 h. Ex vivo permeability: Permeability coefficient: 1.476 ± 0.138 cm/h Permeation flux: 0.148 ± 0.014$\;\mathrm{mg}/{\mathrm{cm}}^{2}h$ In vivo efficacy: demonstrated a 1.7-fold better IOP reduction compared to marketed eye drops.	[61]
To investigate the combination of different grades of Carbopol with HPMC in formulating a sustained release in situ gelling system containing sulfacetamide sodium.	Bacterial conjunctivitis/Sulfacetamide sodium	pH-responsive/Carbopol 940 and HPMC E4M	-	pH: 6.02 Gelation pH: 7.4 (STF) Gelation time: +++ (gel immediately and remained for more than 6–8 h) Viscosity: 1209.00 ± 28.28 cps (gel) In vitro release: cumulative released 90% up to 8 h. In vitro efficacy: demonstrate similar ZOI as marketed eye drops.	[62]
To formulate and characterize the pH-responsive-based hydrogels for effective treatment of the infected eye.	Bacterial conjunctivitis and corneal ulcers/Ofloxacin	pH-responsive/Carbopol and HPMC	-	pH: 6.31 Gelation pH: 7.4 (STF) Gelation time: +++ (gel immediately and remained for more than 8–10 h) In vitro release: cumulative released 97.08% up to 8 h. Ex vivo release: 95.57% of ofloxacin was permeated up to 7 h. In vitro efficacy: demonstrate similar ZOI as standard formulation.	[63]
To evaluate pH-responsive in situ ophthalmic gel system for concomitant delivery of ofloxacin and diclofenac sodium to facilitate sustained release.	Ocular infection and inflammation/Ofloxacin and Diclofenac	pH-responsive/Carbopol 934P and HPMC	Albino rabbits	pH: 6.5 ± 0.2 Gelation pH: 7.2 (STF) Gelation time: +++ (gel within 90 sec and remained for more than 7-8 h) Viscosity: 35 cps (sol) & 1500 cps (gel) Rheology: pseudoplastic flow In vitro release: cumulative released ≈96% of combination drug up to 8 h. Ex vivo permeability: ≈88% of combination drugs permeated for up to 8 h. In vitro efficacy: Demonstrate similar ZOI as standard formulation. Developed in situ gel showed ≥86% antimicrobial effectiveness than the standard solution. In vivo ocular irritation study: no sign of corneal damage.	[64]
To evaluate in situ ocular gel of levofloxacin hydrochloride with increase ocular contact time, enhance the corneal permeability and site-specificity for the better treatment of conjunctivitis and corneal ulceration.	Bacterial conjunctivitis and corneal ulcers/Levofloxacin hydrochloride	pH-responsive/Noveon^®^ AA-1 polycarbophil and HPMC E50LV	Albino rabbits	pH: 6.98 Gelation pH: 7.4 (STF) Gelation time: +++ (gel immediately and remained extended period) Rheology: pseudoplastic flow In vitro release: cumulative released 96.19% up to 8 h. In vitro efficacy: demonstrate similar ZOI as standard formulation. In vivo ocular irritation study: no sign of corneal damage.	[65]
To develop a pH-responsive in situ gel system using moxifloxacin hydrochloride, sodium alginate, and *Terminalia arjuna* gel as gelling agents.	Bacterial conjunctivitis/Moxifloxacin hydrochloride	pH-responsive/Terminalia arjuna and sodium alginate	MCF 7 breast cancer cell line (in vitro cytotoxic) Chorioallantoic membrane (CAM) (ex vivo ocular irritation) Rabbit models (in vivo irritation)	pH: 5.76 Gelation pH: 7.4 (artificial tear solution) Gelation time: ++ (gel immediately and remains for h) Rheology: pseudoplastic flow In vitro release: cumulative released 73.29 ± 1.6% up to 12 h. Ex vivo permeability: demonstrated a 2.3-fold greater amount of permeated drug compared to marketed eye drops. In vitro efficacy: demonstrate similar ZOI as standard formulation. In vitro cytotoxic: displayed negligible cytotoxic effect towards MCF cell lines. Ex vivo ocular irritation study: no toxicity on fertilized eggs. In vivo ocular irritation study: no sign of corneal damage.	[66]
To minimize ocular irritation and prolong the pharmacological action of vancomycin via formulation into nanosized spherical niosome loaded into pH-responsive in situ forming gel.	Ocular infection in MRSA patient/Vancomycin	pH-responsive niosome/Carbopol 934P and HPMC (in situ) with Span 60, Tween 40 and cholesterol (niosome)	Albino rabbits	pH: 5.0 ± 0.2 Gelation pH: 7.4 ± 0.1 (STF) Gelation time: +++ (gel immediately and remained for more than 2 h). Viscosity: 60.8 ± 1.4 Pa·s (gel) Rheology: pseudoplastic flow Mucoadhesion force: 5.2 ± 0.5 Pa In vitro release: cumulative released 39.2 ± 3.2% up to 24 h. In vitro efficacy: Demonstrate similar ZOI as vancomycin solution. Displayed a 2-fold more effective MIC compared to vancomycin solution. In vivo efficacy: showed a 2.5-fold effective in lowered MRSA CFU compared to vancomycin solution. In vivo ocular irritation study: No sign of corneal damage. Histological examination demonstrated that epithelial remained intact.	[67]
To synergize nanoparticle and in situ gel to obtain a norfloxacin formulation with improved residence time and provide sustained release.	Bacterial infection/Norfloxacin	pH-responsive chitosan nanoparticle/Carbopol 934P	CAM (in vitro irritation) Goat cornea (in vivo irritation)	pH: 5.84 ± 0.63 Gelation time: +++ (gel immediately and remained extended period) Rheology: pseudoplastic flow Mucoadhesion force: $1137.45\;dynes/{\mathrm{cm}}^{2}$ In vitro release: cumulative released 88.01 ± 0.48% up to 12 h. In vitro efficacy: demonstrate similar ZOI as marketed eye drops. In vitro ocular irritation study: HET-CAM test showed an irritation score of 0.33 indicated mild irritation. In vivo ocular irritation study: histological examination demonstrated that epithelial remained intact.	[68]
To investigate the amphiphilic block copolymer-based polymeric micellar incorporated in situ ocular gel of itraconazole to manage fungal keratitis.	Fungal keratitis/Itraconazole	pH-responsive polymeric micelle/Carbopol 943P (in situ) with Pluronic-F127 (polymeric micelle)	CAM (in vitro irritation) Goat cornea (in vivo irritation)	pH: 3.20 ± 0.4 (sol) and 6.84 ± 0.34 (gel) Gelation pH: 7.4 (STF) Gelation time: 47.3 ± 4.5 s Mucoadhesion force: 6242.03$\;dynes/{\mathrm{cm}}^{2}$ Ex vivo permeation: Developed micellar in situ gel showed a 5.6-fold higher cumulated drug permeability than itraconazole suspension for up to 8 h. Demonstrated a 3-fold higher permeability flux compared to marketed eye drops. In vitro efficacy: remarkable better ZOI compared to marketed eye drops. In vitro ocular irritation study: HET-CAM test showed an irritation score of 0.67 indicated mild irritation. In vivo ocular irritation study: histological examination demonstrated that epithelial remained intact.	[69]
To develop and evaluate valacyclovir ophthalmic in situ gels based on the concept of pH-responsive in situ gelling systems for the prolonged corneal residence time.	Herpes keratitis/Valacyclovir	pH-responsive/Carbopol 940 and HPMC K100M	-	Gelation pH: 7.4 (STF) Gelation time: +++ (gel immediately and remained extended period) Viscosity: 1090 3.54 cps (gel) Rheology: pseudoplastic flow In vitro release: cumulative released 81.12% up to 8 h.	[70]
To formulate a niosome entrapped in situ hydrogels for sustained release and prolonged the residence time of the formulation.	Herpes keratitis/Acyclovir	pH-responsive niosome/ Carbopol 934 and MC (in situ) with Span 60 and cholesterol (niosome)	Rabbits	Gelation pH: 7.4 (STF) Viscosity: 298 cps (gel) In vitro release: cumulative released 76.5% up to 16 h. In vivo ocular irritation study: no sign of corneal damage.	[71]
To formulate better stability and extensive drug release of naphazoline and antazoline in an in situ gelling systems for ocular allergies.	Allergy conjunctivitis/Naphazoline and Antazoline	pH-responsive/Carbopol 940 and HPMC K4M	Rabbits	pH: 5.75 ± 0.4 Gelation pH: 7.4 (STF) Gelation time: +++ (gel immediately and remained extended period) Viscosity: 10.59 ± 0.34 cps (sol) and 55.96 ± 0.92 cps (gel) Developed in situ gel showed transparent, clear, same gelling capacity and pH value in different storage conditions for 15, 30, and 60 days. In vitro release: cumulative released ≈90% up to 8 h. In vivo distribution: AUC of both drugs was greater compared with marketed eye drops. In vivo ocular irritation study: no sign of corneal damage.	[72]
To develop an optimized formulation of in situ ophthalmic gel of olopatadine hydrochloride by using pH-responsive polymer.	Allergy conjunctivitis/Olopatadine hydrochloride	pH-responsive/Carbopol 974 and HPMC E50LV	Rabbits	pH: 7.0-7.5 Gelation pH: 7.4 artificial tear fluid Gelation time: ++ (gel immediately and remained for 3–4 h) Viscosity: 150.45 cps (gel) In vitro and Ex vivo release: released drug 6 h longer than marketed eye drops. In vivo ocular irritation: no sign of corneal damage.	[73]
To prepare ocular in situ gel of naproxen using carbomer as a pH-responsive gelling agent with different concentrations of a hydrophilic mucoadhesive polymer.	Post-surgery ocular inflammation/Naproxen	pH-responsive/Carbopol 940 and HPMC K100	Rabbits	pH: 5.6 ± 0.02 Gelation pH: 7.4 (STF) Gelation time: +++ (gel immediately and remained extended period) Rheology: pseudoplastic flow. HPMC K100 was favored over HPMC K40 in achieving excellent gelling capacity in combination with Carbopol 940 In vitro release: cumulative release properties for up to 3 h. In vivo ocular irritation: no sign of corneal damage.	[74]
To develop a bear bile-loaded pH-sensitive in situ gel formulation by using a mixture of Carbopol 974 and HPMC K4M.	Glaucoma, Retinitis pigmentosa, age-related macular degeneration and prevent cataract formation/Tauroursodeoxycholic acid (bear bile)	pH-responsive/Carbopol 974 and HPMC K4M	NZW rabbits	pH: 5.0 ± 0.1 Rheology: pseudoplastic flow Residence time: up to 28 min In vitro release: cumulative release properties up to 160 min. In vivo ocular irritation: No sign of corneal damage. Histological examination demonstrated that epithelial remained intact.	[75]
To investigate the correlation between the stability of baicalin and in situ pH-triggered gelling system.	Anti-inflammatory, anti-Chlamydia, anti-bacterial, anti-oxidative, and anti-cataract/Baicalin	pH-responsive/Carbopol 974P and HPMC E4M	NZW rabbits	pH: 5.8 Gelation pH: 6.8 (phosphate buffer) Gelation time: ++ (gel immediately and remained for few h) Rheology: pseudoplastic flow In vitro release: cumulative released ≈95% up to 8 h. In vivo distribution: demonstrated a 6.1-fold greater AUC compared to the control solution. In vivo ocular irritation: no sign of corneal damage.	[76]

**Table 3:** Smart ocular delivery using ion-responsive in situ gel approach against ocular diseases.

Objective	Disease/Drug	Types of Stimuli/Polymer used	Membrane/ Cell Line/ Animal Model	Outcome	Source
To develop a sustained ocular delivery of brinzolamide	Glaucoma/brinzolamide	Ion-activated/deacetylated gellan gum	New Zealand White (NZW) rabbits	pH: 6.32 Gelling capacity: +++ Viscosity: 500 mPa s (solution) and 2200 mPa s (gel) In vitro release: 92% drug release after 12 h. In vivo efficacy: IOP remained lower than baseline after 6 h. In vivo irritation: slight conjunctival hyperaemia in the rabbits.	[87]
To evaluate a sustained release ophthalmic formulation of brinzolamide	Glaucoma/brinzolamide	Ion-activated/deacetylated gellan gum	NZW rabbits	pH: 7 Gelling capacity: +++ Mean residence time: 7.4–17.7 h In vitro release: prolonged-release up to 48 h. In vivo efficacy: Decreased IOP for a longer period than marketed product AUC _(∆IOP vs. t)_ 2–9 times higher than marketed product In vivo irritation: no sign of irritation on NZW rabbits.	[88]
To prepare timolol maleate liposomes dispersed into ion-sensitive in situ ophthalmic gel to enhance drug bioavailability and histocompatibility	Glaucoma/timolol maleate	Ion-activated/deacetylated gellan gum	NZW rabbits	pH: 7.00 Viscosity: 5.07 ± 0.38 mPa s Apparent permeability: 1.93-fold more than eye drops Mean residence time: longer retention time on cornea observed by fluorescence imaging study. In vitro release: sustained release for ≈20 h. In vivo efficacy: Achieved minimum IOP 1 h after use Decreased IOP for 5 h Least IOP increment after induced by water loading. In vivo irritation: TM L-SG showed no significant difference with 0.9% sodium chloride.	[89]
To investigate the therapeutic effectiveness of gatifloxacin ion-activated hydrogel	Bacterial keratitis/gatifloxacin	Ion-activated/gellan gum, sodium alginate	NZW rabbits	High mucoadhesive force In vitro release: retardation of drug release rate In vivo efficacy: Less dosing frequency (twice daily) compared to the marketed formulation (4 times daily) More rapid recovery of bacterial eye infection. In vivo irritation: no sign of irritation on NZW rabbits.	[90]
To evaluate levofloxacin hemihydrate in situ gelation ophthalmic solution	Bacterial conjunctivitis/levofloxacin hemihydrate	Ion-activated gellan gum	NZW rabbits	pH: ≈7.00 Gelling time: <10 s In vitro release: extended-release (18–24 h) In vivo efficacy: 2.7-fold higher ocular bioavailability Maintained levofloxacin MIC for 8–12 h In vivo irritation: no irritation to rabbits’ eyes.	[91]
To fabricate ion-sensitive in situ hydrogels of natamycin (NT)-loaded bilosomes for improved ocular pharmacotherapy	Fungal keratitis/natamycin loaded bilosomes	Ion-activated/gellan gum	Human corneal limbal epithelial cells (HCLE)	pH: 6.4 ± 0.3 Viscosity: 37.5 ± 2.4 cP (solution) and 514.9 ± 3.8 cP (gel) Adhesiveness: 1.39 ± 0.006 g/s In vitro permeation: 6- to 9-fold enhancement in the transcorneal flux. In vivo efficacy: Higher mean dose in cornea Slow-release of bilosomes into ocular tissues. In vitro irritation: cell viability was the same as the negative control.	[92]
Preparation and evaluation of ion-activated in situ gel ophthalmic delivery system of acyclovir based on kappa-carrageenan	Ocular herpes infection/acyclovir- hydroxypropyl-β-cyclodextrin complex	Ion-activated/kappa-carrageenan	NZW rabbits	Rheology: pseudoplastic fluid Gelling capacity: gel rapidly after contacted with tear fluid, maintained for a long time. In vitro release: 80% drug released after 6 h In vitro permeability: 2.16-fold higher apparent permeability. In vivo irritation: no irritation to rabbits’ eyes.	[93]
To formulate an in situ gel for improved residence time and sustained release of nepafenac to the corneal surface	Postoperative corneal pain and inflammation/nepafenac-hydroxypropyl-β-cyclodextrin complex	Ion-activated/sodium alginate	-	pH: 5.63–5.73 In vitro release: sustained drug release over 24 h without burst effect. In vitro permeability: 10 times higher drug penetration (*p* < 0.001) Ex vivo ocular distribution: higher drug retention in cornea, sclera and retina compared to marketed product.	[94]

**Table 4:** Smart ocular delivery using multi-stimuli-responsive in situ gel approach against ocular diseases.

Objective	Disease/Drug	Types of Stimuli/Polymer Used	Membrane/ Cell Line/ Animal Model	Outcome	Source
To characterize and evaluate the transcorneal permeation profile, ocular irritation, and sterility of timolol maleate in pH- and ion-triggered in situ ocular drug delivery system.	Glaucoma/ Timolol maleate	pH-activated/ chitosan and ion-activated/ gellan gum	Goat cornea HET-CAM test New Zealand White (NZW) rabbits	Gelation pH: 6.5–7.0 Viscosity (sol): 40 ± 2.3 cps Viscosity (gel): 150 ± 9.5 cps Have bioadhesive property. Ex vivo permeability assay: improved transcorneal permeation and extended the timolol maleate retention at the corneal site. In vitro ocular irritation (HET-CAM): no irritation. Gamma scintigraphy study: less nasolacrimal drainage and no timolol was observed in systemic circulation.	[96]
To develop and evaluate a combination of chitosan and gellan gum in situ gel of sparfloxacin to lengthen the corneal retention time.	Ocular bacterial infections/ sparfloxacin	pH-activated/chitosan and ion-activated/ gellan gum	HET-CAM test NZW rabbits	Gelation pH: 7.06 ± 0.1 Viscosity (sol): 42.33 ± 1.75 cps Viscosity (gel): 247.33 ± 5.92 cps Have mucoadhesive property. In vitro release study: prolonged release of sparfloxacin. In vitro ocular irritation (HET-CAM): no irritation. Gamma scintigraphy study: better corneal retention.	[97]
To prepare, characterize, and evaluate a combination of chitosan and gellan gum in situ gel formulations for sustained ophthalmic delivery of besifloxacin.	Bacterial conjunctivitis/ besifloxacin	Thermo and pH-activated/ chitosan and ion-activated/ gellan gum	Rabbits’ eye Goat cornea HET-CAM test	Gelation pH: 7.4 Have mucoadhesive properties. In vivo besifloxacin release study: have superior sustain drug release properties. Ex vivo permeation study: enhanced the retention of BSF at corneal surface. In vitro ocular irritation (HET-CAM): no irritation. Histopathology study: no damage seen on corneal membrane. Gamma scintigraphy study: higher amount of BSF retained on the corneal surface. Antimicrobial Study: superior anti-bacterial activity.	[98]
To formulate a combination of chitosan and sodium alginate in situ gel formulation of levofloxacin to prolong contact duration with the ocular surface.	Bacterial keratitis/ levofloxacin	pH-activated/chitosan and ion-activated/ sodium alginate	Albino rabbits	Gelation pH: 7.08 ± 0.07 Viscosity (sol): 52.16 ± 3.48 cps Viscosity (gel): 299.16 ± 29.39 cps Has mucoadhesive properties. In vitro release study: have sustained and control release characteristics. Gamma scintigraphy study: even levofloxacin distribution and none were detected in the systemic circulation.	[99]
To develop combination of Carbopol 940 and gellan gum in situ ophthalmic gel to sustain release of chloramphenicol and dexamethasone sodium phosphate.	Bacterial endophthalmitis/chloramphenicol and dexamethasone sodium phosphate	pH-activated/Carbopol 940 and ion-activated/ gellan gum	-	Viscosity (sol): 50–160 cps Viscosity (gel): 471–6500 cps Mucoadhesive strength: 8.5 ± 0.71–23.6 ± 0.43 g In vitro release study: have sustained release characteristics.	[100]
To design a combination of thermosensitive and ionic sensitive in situ ophthalmic delivery systems to prolong the effect of HP-*β*-CD Voriconazole (VCZ).	Fungal keratitis/ voriconazole	Thermo-activated/Pluronic F-127 or with combination of Pluronic F-68 and ion-activated/ sodium alginate	Goat cornea	Gelation temperature: 24.36 ± 0.41 to 37.33 ± 0.73 °C Viscosity (gel): 235.7 ± 6.66 to 414.3 ± 10.0 cps Mucoadhesive strength: 17.24 to 28.28 dynes/cm^2^ In vitro drug release study and ex vivo permeation study: Slowed down with increasing concentration of each polymer. Anti-fungal efficiency: have prolonged effect and retained its properties against fungal infection.	[101]
To design polymeric nanoparticles of Acyclovir incorporated in in situ gelling system to provide a dual sustained release effect, whereby the duration of action and bioavailability through different routes of administration could be improved.	Ocular viral infection/acyclovir	Thermo-activated/Pluronic F-127 and pH-activated/ Carbopol	-	Gelation temperature: 25 ± 0.20 to 35 ± 0.46 °C Gelation time: 2 to 4 min In vitro drug release study: better sustained release characteristics, with non-Fickian diffusion mechanism of drug release.	[102]
To formulate, optimize, and evaluate the in situ gel for the ophthalmic drug delivery using the combination of gellan gum and Carbopol 934P.	Allergic conjunctivitis/ olopatadine HCl	Thermo-activated/ Carbopol 934P and ion-activated/ gellan gum	NZW rabbits Goat cornea	Viscosity (sol): 34.1 ± 1.2 to 300.7 ± 21.6 cps Viscosity (gel): 471.1 ± 23.5 to 6500.1 ± 234.3 33 cps Mucoadhesive strength: 9.3 ± 0.7 to 33.3 ± 5.2 dynes/cm^2^ In vivo drug release study: sustained olopatadine release Ex vivo permeation study: within desired value range Ocular irritation study and histopathology study: no irritation.	[103]
To formulate capsazepine ocular in situ formulation to treat capsaicin induced ocular tissue inflammation.	Eye irritation cause by capsaicin/ capsazepine	Thermo-activated/ Pluronic F-127 and pH-activated/ chitosan	*Oryctolagus cuniculus* rabbits *Rattus norvegicus* rats Bovine eyes Human Corneal Epithelial Cells and Human Retinal Microvascular Endothelial Cells	Gelation temperature: 28.5 ± 0.62 °C Gelation time: 18 ± 0.68 s In vivo efficacy study: irritation and inflammatory symptoms caused by capsaicin were successfully treated. Ex vivo permeation study: weaker drug permeation. Histopathological study: eased inflammatory ocular responses caused by capsaicin. Cytotoxic study: capsazepine was not cytotoxic in optimum dose.	[104]

**Table 5:** Pros and cons of stimuli-responsive ocular in situ gels.

Type of In-Situ Formulation	Advantages	Disadvantages	Polymer Used
Thermo-responsive	Improved bio-adhesive properties and lengthen the retention time on the corneal site compared to free drug solution, eye drop solution, suspension and single mechanism-based in situ gel formulation Prolong/sustain the duration of drug release compared to eye drop solution, suspension, and nanoparticle formulation Increase the drug retention or permeation on the corneal surface compared to free drug solution, suspension, and single mechanism based in situ gel formulation Promotes even drug distribution on the corneal surface and lessens the nasolacrimal drainage of the drug into the systemic circulation compared to free drug solution, eye drop solution, and single mechanism based in situ gel formulation	Not suitable to be having around in hotter climate countries, Leakage of gel may occur if there is no immediate phase transition from sol to gel.	Poloxamer, PNIPAAM, cellulose derivatives
pH-responsive		Stability of basic drugs may be challenging as pH-sensitive polymers used are acidic Sudden shift of pH may cause mild irritation of the eyes. Leakage of gel may occur if there is no immediate phase transition from sol to gel.	Carbomer-910, -934, -941, -940, PCP, Chitosan
Ion-responsive		Leakage of gel may occur if there is no immediate phase transition from sol to gel.	Carrageenan, gellan gum, alginate
Multi-stimuli-responsive		All of the above	All polymers as mentioned above

## Data Availability

Data is freely available.
